# Enhanced eddy activity in the Beaufort Gyre in response to sea ice loss

**DOI:** 10.1038/s41467-020-14449-z

**Published:** 2020-02-06

**Authors:** Thomas W. K. Armitage, Georgy E. Manucharyan, Alek A. Petty, Ron Kwok, Andrew F. Thompson

**Affiliations:** 1 Jet Propulsion Laboratory, California Institute of Technology, Pasadena, CA USA; 2 School of Oceanography, University of Washington, Seattle, WA USA; 3 Cryospheric Sciences Laboratory, NASA Goddard Space Flight Center, Greenbelt, MD USA; 4 Earth System Science Interdisciplinary Center, University of Maryland, College Park, MD USA; 5 Environmental Science and Engineering, California Institute of Technology, Pasadena, CA USA

**Keywords:** Cryospheric science, Physical oceanography

## Abstract

The Beaufort Gyre freshwater content has increased since the 1990s, potentially stabilizing in recent years. The mechanisms proposed to explain the stabilization involve either mesoscale eddy activity that opposes Ekman pumping or the reduction of Ekman pumping due to reduced sea ice–ocean surface stress. However, the relative importance of these mechanisms is unclear. Here, we present observational estimates of the Beaufort Gyre mechanical energy budget and show that energy dissipation and freshwater content stabilization by eddies increased in the late-2000s. The loss of sea ice and acceleration of ocean currents after 2007 resulted in enhanced mechanical energy input but without corresponding increases in potential energy storage. To balance the energy surplus, eddy dissipation and its role in gyre stabilization must have increased after 2007. Our results imply that declining Arctic sea ice will lead to an increasingly energetic Beaufort Gyre with eddies playing a greater role in its stabilization.

## Abstract (web summary)

The freshwater content of the Beaufort Gyre in the Western Arctic Ocean has increased in response to almost two decades of persistent anti-cyclonic winds. Here, the authors found that dramatic loss of sea ice and acceleration of surface currents after 2007 led to a net annual wind energy input to the Beaufort Gyre, and anticipate that continued sea ice decline will lead to an increasingly energetic Beaufort Gyre.

## Introduction

**Fig. 1:**
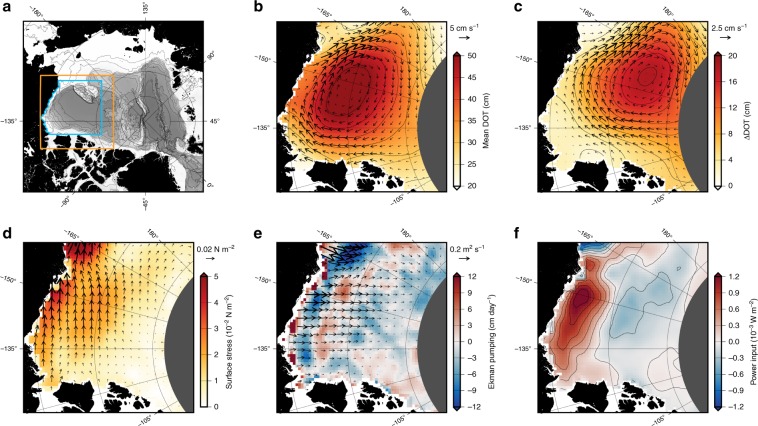
State of the Beaufort Gyre 2003–2014. **a** A map of the Arctic Ocean showing the Western Arctic region plotted in **b**–**f** (orange line), the Beaufort Gyre region over which area averages are computed (blue line) and 500 m isobaths taken from Amante and Eakins^44^ (black lines). **b** The 2003–2012 mean dynamic ocean topography (shading; contours are drawn every 5 cm) and surface geostrophic currents (cm s^−1^; vectors). **c** The change in dynamic ocean topography (shading; vectors drawn every 2 cm) and geostrophic currents (cm s^−1^; vectors) between 2003–2006 and 2008–2014, **d** the 2003–2014 mean ocean surface stress (N m^−2^; vector and shading), **e** the 2003–2014 mean Ekman pumping velocity (shading) and Ekman layer velocity (m^2^ s^−1^; vectors), and **f** the 2003–2014 mean wind power input.

The Beaufort Gyre (BG) is an anticyclonic sea ice–ocean circulation system, driven by the semipermanent Beaufort Sea high-pressure system, and is the dominant sea ice and ocean surface circulation feature of the western Arctic Ocean (Fig. 1a, b)^1,2^. Negative wind stress curl over the region (Fig. 1d), mediated by the sea ice pack, leads to Ekman convergence (Fig. 1e), downwelling of isopycnal surfaces, and storage of ~20,000 km^3^ of freshwater in the upper few hundred meters of the ocean^3,4^. During a period of dramatic environmental change, including the rapid loss of sea ice, in particular older and thicker perennial ice^5–7^, the BG has accumulated ~8000 km^3^ of freshwater since the 1990s and ~5000 km^3^ since the early 2000s^3,4,8–11^. The accumulation of low-density surface water is reflected by increased sea level^8,11,12^ and doming of the dynamic ocean topography, resulting in increased surface geostrophic circulation (Fig. 1c)^8,10,13^. The majority of past studies of the BG have focused on understanding changes in BG freshwater content (FWC) in relation to linear Ekman dynamics^3,8,10,14–19^. However, the time-mean area-averaged Ekman pumping across the BG is persistently negative, implying a tendency for halocline deepening and freshwater accumulation that cannot continue in perpetuity and must ultimately be balanced by other processes.

Recently, two plausible hypotheses regarding the internal mechanisms of BG FWC stabilization have emerged. The first hypothesis relies on the fact that the ice–ocean surface stress depends on the vector difference between the sea ice and ocean surface velocities (see “Methods”), such that an intensification of geostrophic currents due to surface freshwater accumulation and steepening of the dynamic ocean topography tends to reduce the sea ice–ocean surface stress, limiting further Ekman pumping in a negative feedback mechanism. The sea ice–ocean stress feedback mechanism has been termed the ice–ocean governor^20^. Indeed, it has been shown that including time-variable surface geostrophic currents in sea ice–ocean surface stress calculations reduces the net Ekman pumping in the BG by as much as 50–70% relative to a stationary ocean^14,15,19^. The second hypothesis emphasizes that increased Ekman pumping, freshwater storage, and steepening of the BG halocline slope is associated with generation of available potential energy (APE; the gravitational potential energy stored in a sloped halocline), and that this should be counteracted by increased production of eddies by baroclinic instabilities. Halocline eddies then cumulatively act to flatten isopycnal slopes, dissipating APE, weakening geostrophic currents, and diffusing freshwater by lateral mixing^15,21–25^. Consistent with this hypothesis, an increased number of halocline eddies has been observed in recent years^26^, and observational estimates of eddy diffusivities from BG moorings were found to be sufficiently strong to counteract Ekman-driven freshwater accumulation^27^. However, due to the relative scarcity of data, broad-scale observational evidence of the eddy stabilization hypothesis has yet to be demonstrated. In reality, despite the role of the ice–ocean governor, there is still a significant net downwelling in the BG region (Fig. 1e), so understanding the evolution and stabilization of BG FWC requires an assessment that accounts for both hypotheses.

Here, we provide insight into BG FWC stabilization and energy dissipation mechanisms by considering the oceanic mechanical energy budget. Energy budget considerations have been important for understanding dynamics of the global ocean (e.g., Ferrari and Wunsch^28^), however this approach has been limited in the polar oceans by a lack of data. Using satellite-derived estimates of surface geostrophic currents underneath sea ice^11,13^, combined with reanalysis wind, satellite-derived sea ice drift and concentration, and in situ hydrographic data, we present the first observational estimates of the BG wind energy input and potential energy sources and sinks over monthly to interannual time scales. Our results reveal a delicate balance between wind energy input in seasonally ice-free BG regions, energy dissipation underneath sea ice, APE storage, and energy dissipation by eddies, which is evolving over time as the Arctic sea ice pack recedes under climate change.

## Results and discussion

### Beaufort Gyre energy sources and sinks

**Fig. 2:**
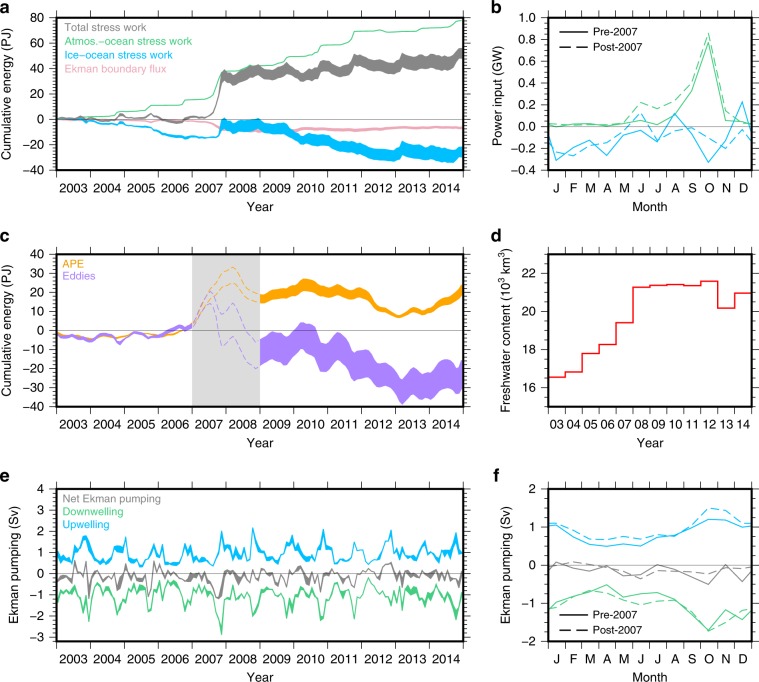
Beaufort Gyre Energy and Freshwater Budget. **a** The atmosphere-ocean (green), ice–ocean (blue), and net (atmosphere-ocean + ice-ocean; gray) cumulative energy input to the Beaufort Gyre region (gray), and the boundary thickness fluxes due to Ekman transport (pink). **b** The monthly ice–ocean (blue) and atmosphere-ocean (green) seasonal cycle of power input before (solid lines) and after (dashed lines) 2007. **c** The available potential energy (*APE*, orange) and cumulative eddy dissipation (purple) in the Beaufort Gyre region, and **d** the Beaufort Gyre liquid freshwater content estimates^3^. **e** The Ekman upwelling (blue), downwelling (green), and net pumping (gray) in the Beaufort Gyre region, and **f** the seasonal cycle of Ekman pumping before (solid lines) and after (dashed lines) 2007. The gray box in **c** corresponds to the period when the relationship between DOT and halocline depth breaks down and our estimates of APE and eddy dissipation become unreliable (see “Methods”). The spread on the data in **a, e** represents the difference in the calculation of wind energy input and Ekman pumping using two different sea ice drift data sets, and the spread in **c** also incorporates the uncertainty of the fit between dynamic ocean topography and halocline depth (see “Methods”).

The mechanical energy budget is formulated by considering the depth evolution of the halocline to derive an expression for the rate of change of APE in the BG region (see “Methods”). The terms include wind energy input (i.e., the work done by the ocean surface stress on the surface geostrophic circulation), boundary thickness fluxes due to Ekman transport across open lateral boundaries, and eddy diffusion. To estimate these terms, we calculate the ocean surface stress from reanalysis wind, satellite-derived sea ice drift and concentration, and estimates of surface geostrophic currents produced by combining conventional radar altimetry data from the open ocean with specialized altimetry retrievals from openings within the sea ice pack^11,13^. This allows us to calculate the wind work and the cumulative wind energy input to the BG region (Fig. 2a, b), as well as the Ekman pumping (Fig. 2e, f). We make use of in situ hydrographic data to estimate the difference in density across the halocline, and use the relationship between the hydrography-derived halocline depth and DOT to estimate halocline depth across the BG region. This allows us to estimate the APE and boundary thickness fluxes (Fig. 2a, c) as well as the kinetic energy (KE) of the geostrophic circulation, which is negligible relative to the other terms. The residual of the wind energy input, Ekman boundary fluxes, and APE storage then represents the energy dissipation by eddies (Fig. 2c).

**Fig. 3:**
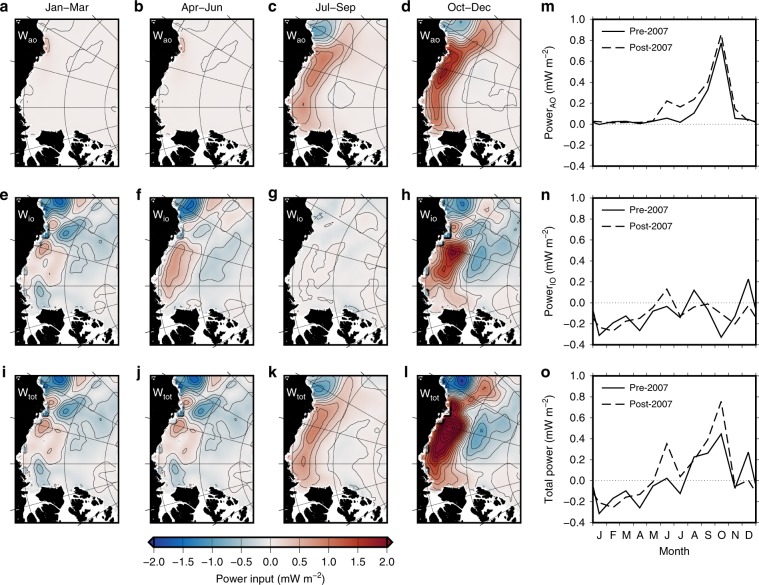
Seasonal wind energy input to the Beaufort Gyre. Seasonal climatologies of the atmosphere-ocean (**a**–**d**), ice–ocean (**e**–**h**), and total (**i**–**l**) wind energy input for winter (January–March), spring (April–May), summer (June–September), and autumn (October–December), as well as the mean seasonal cycles (**m**–**o**) before and after 2007.

Wind energy input to the BG is highly seasonal, spatially inhomogeneous, and heavily dependent on the sea ice cover and atmospheric circulation (Figs. 2b and 3). The BG gains energy from the winds in the south, and loses energy in the north over a mean annual cycle (Fig. 1f). Strong atmospheric circulation in the autumn, combined with significant areas of open water, means that work done by the atmosphere directly on the surface geostrophic currents (*W*_ao_) dominates energy input to the BG. Around 60% of the total wind power input occurs in September and October in the ice-free southern BG, where easterly along-shore winds do work on the westward flowing southern limb of the BG (Fig. 3). Averaged over the BG region, sea ice acts to dissipate energy in most months (Fig. 2b), dominated by negative ice–ocean surface stress work (*W*_io_) in the northern BG region during winter (Fig. 3). This is the ice–ocean governor from the perspective of mechanical energy: the upper ocean flows beneath a relatively immobile ice pack and undergoes top-boundary drag, which dissipates energy while acting to spin down the gyre by inducing cyclonic stress and Ekman upwelling^14,15,19^. Ekman pumping is also at its seasonal maximum in the autumn (Fig. 2f), corresponding to the peak in the seasonal cycle of freshwater storage^3,11^.

**Fig. 4:**
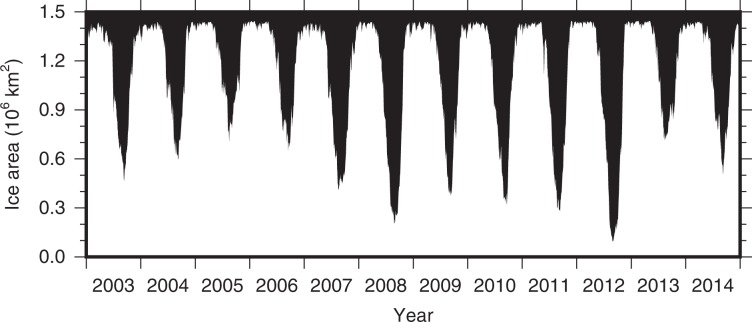
Area of sea ice. Time series of sea ice area (10^6^ km^2^) in the Beaufort Gyre region corresponding to the blue box in Fig. 1a.

Between 2003 and 2006, wind energy input was balanced by energy dissipation under sea ice over an annual cycle and there was not a significant net energy input to the BG region (Fig. 2a). At the same time, there was no significant change in APE during this period, implying that the role of eddies in dissipating energy was small. During this period, the BG can be thought of as existing in an “energetic governor regime”, where wind energy input was balanced by energy dissipation under sea ice and there was little change in APE storage. This corresponded to a period of net Ekman downwelling of ~170 mSv (Fig. 2e; 1 mSv ≡ 10^3^ m^3^/s) and an increase in BG FWC of almost 2000 km^3^ over 4 years (Fig. 2d). However, this balance changed dramatically in late 2007, when a strong wind event injected ~30 PJ of wind energy into the BG region (Fig. 2a). Large open water areas associated with the then-record summer 2007 minimum sea ice extent^7^ (Fig. 4) meant that strong and persistent anticyclonic wind anomalies over the western Arctic did a large amount of work on surface currents in the southern BG that were flowing at least twice as fast as the climatological average^13^. FWC and APE in the BG increased significantly in response to this forcing (Fig. 2c, d) implying a potential for enhanced eddy generation by instabilities acting on the increased isopycnal slope. However the eddy response may lag the forcing by a few years^24^.

After 2007, wind energy input and energy dissipation underneath sea ice both intensified and became more seasonal (Fig. 2a, b). Diminished summer and autumn ice cover (Fig. 4) led to higher wind energy input and generally less energy dissipation by sea ice between June and November; at the same time, increased surface currents in winter resulted in greater energy dissipation by sea ice between December and March (Fig. 2b). Between 2009 and 2014, there was a total wind energy input of ~9.5 PJ, the Ekman boundary flux added ~2.8 PJ, while APE decreased by ~3.2 PJ (Fig. 2a, c). We estimate ~15.7 PJ of energy dissipation by eddies between 2009 and 2014. After 2007, the ice–ocean governor mechanism was no longer able to fully dissipate the increased wind energy input, and the BG mechanical energy budget was out of equilibrium. Ekman upwelling and downwelling both increased after 2007, which will tend to produce a steeper halocline slope (Fig. 2e, f), however the net Ekman pumping actually decreased after 2007 (Fig. 2e, f) and the FWC remained relatively stable from 2008 onwards (Fig. 2d). We note that, as we use the 12-month smoothed DOT to estimate the halocline depth (see “Methods”), our estimates of APE, and hence eddy dissipation, break down during periods of rapid change (i.e., late 2007 ± ~1 year). This occurs because DOT varies faster than the halocline depth and the correlation between the two is weaker at short (~monthly) time scales.

### Eddies role in Beaufort Gyre energy and freshwater balance

From energy budget considerations, the increased wind energy input to the BG after 2007, and the reduction in the APE reservoir after 2007, implies that energy dissipation associated with eddies must also have increased after 2007. The surface geostrophic currents data resolve ocean variability to a resolution of O (80 km), at monthly time scales^13^, leaving unresolved the energetic transient eddies with a characteristic scale of the Rossby radius, which is ~10–15 km in the Canada Basin^29^. There are several ways that transient surface eddies could contribute to increased energy dissipation. The cubic dependence of ice–ocean surface stress work on the characteristic eddy velocity, *u*_eddy_, means that energy dissipation by eddies is highly sensitive to small changes in *u*_eddy_. Changes in the ice–ocean drag coefficient, due to, e.g., changes in form drag^30^, or an increase in eddy density^26^, will also affect energy dissipation underneath sea ice. The spatial pattern of wind energy input to the BG (input along the southern edge, dissipation in the interior; Fig. 1f) implies a northward transport of energy from source to sink. Our method does not allow us to investigate this in detail and we can only speculate that the energy is transported by the mean geostrophic circulation as well as by preferential mesoscale eddy propagation away from the southern BG region, where strong baroclinic currents near the gyre boundaries facilitate eddy formation, towards the interior of the gyre where there is mechanical dissipation. As the eddies propagate to the interior of the gyre, they transport freshwater anomalies (halocline thickness anomalies) but also the eddy APE and eddy KE. Observations and theory of baroclinic ocean turbulence in the ice-free global ocean suggest that bottom drag is a key energy dissipation mechanism (e.g., Ferrari and Wunsch^28^). However, the Western Arctic is highly baroclinic, and currents at depth are an order of magnitude weaker than at the surface^31^, implying bottom drag work of order 10^−3^–10^−2^ GW, two or three orders of magnitude smaller than the surface energy input/dissipation (Fig. 2b). Hence, we conclude that an enhancement of upper-ocean turbulence, specifically the generation of mesoscale eddies, is critical to balance the increased wind-driven energy input into the BG.

**Fig. 5:**
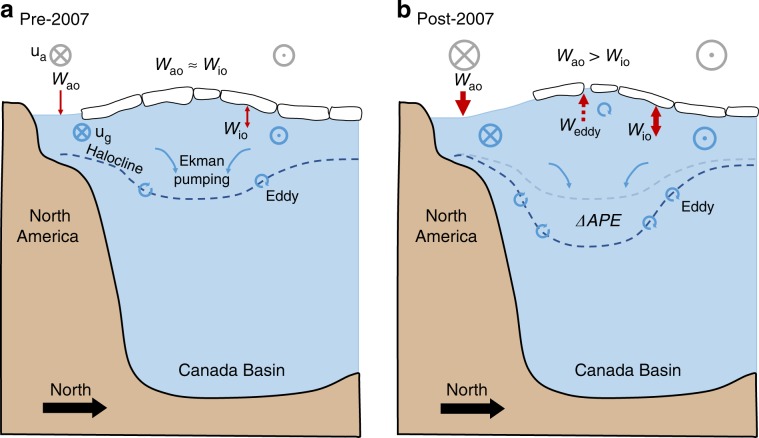
The changing components of the Beaufort Gyre energy budget. **a** Before and **b** after 2007, including the wind work, *W* (comprised of atmosphere-ocean, *W*_ao_, and ice–ocean, *W*_io_, components), available potential energy (APE), and eddy dissipation, *W*_eddy_. The atmosphere and ocean circulations are illustrated by *u*_a_ and *u*_g_, respectively. The size of the arrows/vectors represents their relative strength. The loss of sea ice after 2007 led to increased wind energy input to the BG, increased APE, and increased energy dissipation and freshwater stabilization by eddies.

Analogous to increased energy dissipation, the continued net Ekman downwelling after 2007 coupled with the stability of BG FWC after 2008 (Fig. 2d–f) implies a greater role for eddies in the FW budget. For the ice–ocean governor mechanism to fully compensate for increased Ekman pumping^14,15,19^, geostrophic currents must increase until Ekman upwelling, induced by ocean currents flowing beneath slower moving sea ice, fully compensates the Ekman downwelling. However, this is not the case in the Arctic after 2007. While the net Ekman downwelling does decrease after 2007, implying a role for an increased ice–ocean governor mechanism, increased halocline eddy activity must also play a role for the BG FWC to stabilize. From the expression for the eddy dissipation term (see “Methods”) we can estimate a value for *K*_GM_, the eddy diffusivity, of ~120 m^2^/s for the period after 2008 when eddies are found to play an important role in energy dissipation. This falls within the range of values estimated by Meneghello et al.^27^, and represents a gyre-wide mean that could be expected to vary locally and in time. The energy-budget approach together with considerations of Ekman-driven freshwater accumulation and release consistently reveal an increasing role for eddies in energy dissipation and FWC stabilization in the BG, particularly from 2008 onwards. Overall, the picture is one of a more energetic BG system since 2007, with increased energy sources and sinks and increased eddy diffusivity balancing and dissipating APE, and FWC stabilization by eddies required to balance net Ekman downwelling (Fig. 5).

### Implications for the changing Arctic

Arctic sea ice loss is projected to continue over the coming decades, with climate models predicting seasonally ice-free conditions (<10^6^ km^2^) as early as the 2020s, but more likely towards the middle of the century^7,32^. Previous hypotheses suggested that the Arctic atmospheric circulation oscillates between predominantly cyclonic and anticyclonic circulation regimes over timescales of 5–7 years^1^, however, the Arctic has been in an anticyclonic regime since the late 1990s, coinciding with a period of increasing freshwater storage^9^. Were the Arctic to switch back to predominantly cyclonic atmospheric circulation we might expect a dissipation of mechanical energy by the atmosphere in summer and autumn months, as well as weaker (or reversed) net Ekman pumping and release of freshwater. Here, the results from 2012 provide a useful test case in contrast to the extreme of 2007. In 2012, as in 2007, there was an almost complete loss of sea ice in the BG region (Fig. 4), the cyclonic atmospheric circulation conditions actually dissipated energy in the summer (Fig. 2a) and were more favorable for upwelling (Fig. 2e) causing a (temporary) reduction in FWC (Fig. 2d). The difference in the BG response during extreme ice loss in 2012 compared to 2007 highlights the important interplay between atmospheric circulation and sea ice conditions that controls the state of the BG. A similar reversal of the prevailing anticyclonic atmospheric circulation was observed in wintertime 2016–17. This event was linked to increased intrusions of Atlantic cyclones due to thin ice in the Barents Sea region (and associated thermal anomalies), and a shift in the polar vortex^33^. Increased intrusions of cyclones into the western Arctic and further reversals in the wintertime atmospheric circulation due to declining sea ice in the Barents Sea is an intriguing, but highly uncertain, hypothesis^34^. These events suggest that a switch to more cyclonic circulation conditions would lead to a period of freshwater release and a spin down of the BG. However, regardless of the prevailing atmospheric circulation regime, we expect the Arctic Ocean to become more sensitive to atmospheric forcing as the sea ice cover continues to decline under climate change.

Our results show that as the BG region becomes increasingly ice-free earlier in summer and later into October and November, the current anticyclonic atmospheric circulation regime will do significantly more work on the ocean surface currents. Meanwhile, year-round dissipation of energy underneath sea ice will also increase as currents speed up (Fig. 2a, b), but our results suggest that this will not completely account for increased direct atmosphere-ocean energy input. Under this scenario, the Arctic Ocean will continue to become more energetic, and dissipation of additional energy and freshwater stabilization by eddies will be increasingly important. These are critical processes for the accurate representation of the BG system in models. However, currently, only the highest resolution numerical models are eddy-resolving in the Arctic Ocean, where the radius of deformation is 10–15 km in the deep basins and as small as 1 km in the shelf seas. Coupled climate models leave these important dissipative processes unresolved and it is unclear whether commonly used parameterizations of eddies, tuned for the global ocean, are representative in the Arctic. Increases in the eddy diffusivity and increased mixing by eddy activity in a more energetic Arctic Ocean is also expected to enhance vertical transport of warm Atlantic water, with consequences for sea ice growth and mixing of biogeochemical tracers.

## Methods

### Available potential energy budget

We define the APE as the difference between the gravitational potential energy and its reference state obtained by adiabatic flattening of the halocline depth:

1 $$ {{\rm{APE}}\,=\,{\rm{PE}}\,-\,{\rm{PE}}_{{\mathrm{ref}}}\,=\,\frac{1}{2}g\Delta \rho {\int\!\!\!\int} {\left( {h^2\,-\,\bar h^2} \right)} dA} ,$$

where the reference potential energy ${\rm{PE}}_{{\mathrm{ref}}}\,=\,0.5Ag\Delta \rho \bar h^2$, $\bar h\,=\,A^{ - 1}\smallint \smallint hdA$, and *A* is the gyre area, after Gill et al.^35^. Note that the reference APE state determined by the spatial mean halocline depth can evolve in time and this must be considered when considering the APE evolution.

Since the APE depends on halocline perturbations from the horizontal mean, we consider the halocline depth evolution equation:

2 $$ {\frac{{\partial h}}{{\partial t}}\,+\,{\mathrm{u}}_{\mathrm{g}} \cdot \nabla h\,=\,K_{{\mathrm{GM}}}\nabla ^2h\,-\,w_{{\mathrm{Ek}}}} ,$$

where *w*_Ek_ is defined positive upwards (upwelling decreases *h*), *K*_GM_ is the eddy diffusivity, and **u**_g_ is the surface geostrophic current. Since in our framework the geostrophic current components can be written as:

3 $$ {u_{\mathrm{g}}\,=\,-\frac{{g\Delta \rho }}{{f\rho _{\mathrm{w}}}}\frac{{\partial h}}{{\partial y}};v_{\mathrm{g}}\,=\,\frac{{g\Delta \rho }}{{f\rho _{\mathrm{w}}}}\frac{{\partial h}}{{\partial x}}} ,$$

(see the halocline depth derivation below) the term ${\mathbf{{u}}}_{\mathrm{g}} \cdot \nabla h\,=\,0$, and only advection by ageostrophic circulation can lead to halocline thickness tendencies. Here we only consider large spatial and temporal scales so we only keep the Ekman components of the ageostrophic circulation. In this formulation, the APE (Eq. 1) and geostrophic currents (Eq. 3) are both diagnostic variables of the halocline depth, *h*, however the relation between APE and geostrophic currents can be nontrivial and they are not necessarily expected to be linearly correlated. This is because the APE is defined as a “global” quantity which depends on the halocline depth anomaly from the state of rest, i.e., it depends on the spatial distribution of the halocline across the entire domain. The geostrophic current, on the other hand, is defined as a local quantity that depends on the local gradient of the halocline depth and does not depend on the halocline depth distribution elsewhere in the domain.

Multiplying the halocline depth evolution by *h* and integrating over the gyre we obtain (after some algebra):

4 $$\frac{{\partial {\rm{APE}}}}{{\partial t}}\;=\; 	\,K_{{\mathrm{GM}}}g\Delta \rho {\int\!\!\!\int} {\left( {h - \bar h} \right)} \nabla{^2} hdA\, +\,{\int\!\!\!\int} {\left( {{\uptau }}_{\mathrm{o}} \cdot {\mathbf{{u}}}_{\mathrm{g}} \right)} dA\\ 	\, -\,\frac{{g\Delta \rho }}{{\rho _wf}}\mathop {\iint}\nolimits_\Gamma {\left( {h\,-\,\bar h} \right)} \left( {{\uptau} _{\mathrm{o}} \cdot d{\mathrm{l}}} \right) ,$$

where *d*l is the vector length element positive in the cyclonic direction along the gyre boundary Γ. The rate of change of the APE is therefore determined by the eddy term (first term on the RHS), the wind work (second term on the RHS), and boundary thickness fluxes due to Ekman transport (third term on the RHS). We write the total wind work as

5 $${W_{{\mathrm{tot}}}\,=\,{\int\!\!\!\int} {\left( {{\uptau }}_{\mathrm{o}}\,\times\,{\mathbf{{u}}}_{\mathrm{g}} \right)} dA},$$

or, alternatively:

6 $${W_{{\mathrm{tot}}}\,=\,W_{{\mathrm{ao}}}\,+\,W_{{\mathrm{io}}}\,=\,{\int\!\!\!\int} {\left( {1\,-\,A_{\mathrm{i}}} \right)} \left( {{\uptau} _{{\mathrm{ao}}} \cdot {\mathbf{u}}_{\mathrm{g}}} \right)dA\,+\,{\int\!\!\!\int} {A_{\mathrm{i}}} \left( {{\uptau} _{{\mathrm{io}}} \cdot {\mathbf{u}}_{\mathrm{g}}} \right)dA} ,$$

where *A*_i_ is the sea ice concentration. Here, we estimate the APE (from Eq. 1), wind work, and Ekman boundary flux terms, allowing us to estimate the eddy term as the residual in Eq. (4). As discussed in Roquet et al.^36^, the overall energy input, *W*_tot_, is partially redistributed laterally within the Ekman layer before entering the gyre interior, however we are focusing on the area-integrated energy balance, and refer readers to Roquet et al.^36^ for more discussion.

### Ocean surface stress and Ekman pumping

We compute the daily ocean surface stress, **τ**_o_, as the ice concentration-weighted mean of the atmosphere–ocean and ice–ocean stress, after^20^:

7 $$\uptau _{\mathrm{o}}\,= 	\,\left( {1\,-\,A_{\mathrm{i}}} \right)\uptau _{{\mathrm{ao}}}\,+\,A_{\mathrm{i}}\uptau _{{\mathrm{io}}}\,=\,\left( {1\,-\,A_{\mathrm{i}}} \right)C_{{\mathrm{dao}}}\rho _{\mathrm{a}}{\mathbf{{u}}}_{\mathrm{a}}\left| {{\mathbf{{u}}}_{\mathrm{a}}} \right|\\ 	\, +\,A_{\mathrm{i}}C_{{\mathrm{dio}}}\rho _{\mathrm{w}}( {{\mathbf{{u}}}_{\mathrm{i}}\,-\,{\mathbf{{u}}}_{\mathrm{g}}} )\left| {{\mathbf{u}}_{\mathrm{i}}\,-\,{\mathbf{u}}_{\mathrm{g}}} \right|e^{i\beta } ,$$

where *C*_dao_ and *C*_dio_ are the atmosphere–ocean and ice–ocean drag coefficients, *ρ*_a_ = 1.25 kg m^−3^ and *ρ*_w_ = 1024 kg m^−3^ are the atmosphere and upper ocean densities, **u**_a_, **u**_i_, and **u**_g_ are the vector wind, ice, and geostrophic ocean current velocities and *β* = 23° is the ice–ocean turning angle. We take the wind-speed dependent form of *C*_dao_ from Large and Pond^37^, and use the standard values of *C*_dio_ = 0.0055 (e.g., Meneghello et al.^15^). The Ekman pumping is then:

8 $${w_{{\mathrm{Ek}}} = \frac{1}{{\rho _{\mathrm{w}}f}}\nabla\, \times\, {\uptau}_{\mathrm{o}}} ,$$

where *f* is the Coriolis frequency.

To make the above calculations we average 6-hourly analyzed 10 m vector wind fields from the ERA-Interim Reanalysis to form daily mean wind fields^38^. Petty et al.^39^ analyzed the wind field curl in the BG region from multiple reanalyses (ERA-Interim, NCEP-R2, JRA-55), and found strong consistency in the magnitude and interannual variability of the different products. We perform the surface stress calculations using two different sea ice motion data sets: the 25 km Polar Pathfinder (PP) data archived by NSIDC^40^ and data derived from Advanced Microwave Scanning Radiometer (AMSR)-E and AMSR-2 passive microwave data, described by Kwok et al.^41^. It is known that the PP data are biased slow (e.g., Sumata et al.^42^) and performs less well in proximity to the coast compared with the Kwok et al.^41^ data. However, we require the continuous daily coverage provided by the PP data, since there was a gap in the AMSR record between October 2011 and July 2012 that we fill with the PP data. We show the calculations using both sea ice motion data records to demonstrate the spread introduced by using different ice drift data (Fig. 2). Arctic Ocean surface geostrophic currents were taken from Armitage et al.^13^. Since higher temporal resolution is not currently possible, monthly currents were interpolated in time to obtain daily currents. Both the dynamic ocean topography data used to derive the currents, and the derived currents data themselves, show good agreement with in situ data^11,13^. Daily sea ice concentration are the 25 km NASA Bootstrap data archived by NSIDC^43^. All data were projected on the 25 km SSM/I polar stereographic grid documented by the National Snow and Ice Data Center (NSIDC, nsidc.org/data/polar-stereo/ps_grids.html). Area-weighted means were calculated over the BG region shown in Fig. 1a.

### Halocline properties

To estimate the terms in the energy budget we require spatially extensive estimates of the halocline depth, *h*, and the change in density across the halocline, Δ*ρ*. To first order, we expect the halocline depth to be related to the dynamic ocean topography, *η*, by

9 $${h\,=\,\frac{{\rho _{\mathrm{o}}}}{{\Delta \rho }}\eta\,+\,\varepsilon },$$

where *ρ*_o_ is the ocean density at the bottom of the halocline and *ε* accounts for any static offset in the reference geoid used to estimate *η*. We estimate *h* and Δ*ρ* using hydrographic profiles collected by year-round moorings and annual CTD surveys in the BG region since 2003 as part of the Beaufort Gyre Exploration Project (http://www.whoi.edu/website/beaufortgyre/home). The bottom of the mixed layer is detected as the first pressure bin in which the salinity deviates by 0.1 from the mean salinity in the upper three bins (Supplementary Fig. S1a). The bottom of the halocline is estimated as the depth bin corresponding to the largest peak in the derivative of density with respect to depth, for depths greater than 100 m (Supplementary Fig. S1b). The halocline depth, *h*, is then simply the depth of the halocline bottom with respect to the surface, and we take Δ*ρ* as the difference between the mean density in the halocline layer (taken as the bottom of the mixed layer to the bottom of the halocline) and the mean density in the 200 m immediately below the halocline (Supplementary Fig. S1c). Throughout the study period, *h* varies in the BG region, spatially and temporally, between 130 and 230 m and Δ*ρ* between 2.5 and 3.5 kg/m^3^. In the APE calculation we simply take the mean value of Δ*ρ*. We find the linear regression between *h* and the 12-month smoothed DOT as we do not expect the halocline depth to vary as quickly as the DOT; we find good correlation between *h* and the smoothed DOT (Supplementary Fig. S4; *R* = 0.83). Application of a 12-month running mean introduces a paradox, in that the estimated halocline depth depends on the DOT 6 months in the future, so we focus our discussion on multiannual changes (i.e., from 2009 to 2014, much longer than the 12-month window) rather than computing linear trends which may be misleading. While the application of the 12-month smoothing increases the correlation between DOT and halocline depth, the length of the smoothing window does not significantly affect the conclusions of the study. The derived regression coefficients are used to estimate *h* from *η* across the entire BG region for every month of the study period (Supplementary Figs. 2 and 3). Note that the use of smoothed DOT means that the estimated halocline depth (and hence energy terms) is likely inaccurate during periods of rapid change (i.e., 2007 in our study), and results in these time periods should be treated with caution.

### Kinetic energy

The KE of the monthly mean geostrophic flow also contributes to the total energy budget. We compute the KE the BG region as:

10 $${{\rm{KE}}\,=\,\frac{1}{2}\rho \iint_{{\mathrm{BG}}} {dA} \int_{ - {\mathrm{h}}}^0 {u_{{\mathrm{g}}}} \left( {x,y,z} \right)^2\,+\,v_{\mathrm{g}}\left( {x,y,z} \right)^2dz} ,$$

where *ρ* is the ocean density, the area integral is performed over the BG region (Fig. 1a), the depth integral is performed from the halocline depth, *h*, to the surface, and we assume that **u**_g_ and **v**_g_ vary linearly from the geostrophic current speed at the surface to zero at *h*. Note that, if anything, this assumption will overestimate the KE of the geostrophic flow, as generally the geostrophic current will decay more quickly with depth. However, the KE is much smaller than the APE and can be disregarded from the rest of the calculations (Supplementary Fig. 4), with |*APE*/*KE*| > 100 in general. Note that it is generally the case that KE is negligibly small compared with APE, as noted by Gill et al.^35^.

## Supplementary information

Peer Review File

Supplementary Information

## Data Availability

Six-hourly analyzed 10 m vector wind fields were taken from the ERA-Interim Reanalysis^38^ and are available via https://apps.ecmwf.int/datasets/data/interim-full-daily/levtype=sfc/. Sea ice motion was taken from the 25 km Polar Pathfinder (PP) data archived by NSIDC^40^ (available via https://nsidc.org/data/nsidc-0116/) and from Advanced Microwave Scanning Radiometer (AMSR)-E and AMSR-2 passive microwave data, described by Kwok et al.^41^ and available upon request. Arctic dynamic ocean topography and geostrophic currents data were provided by the Centre for Polar Observation and Modelling, University College London (www.cpom.ucl.ac.uk/dynamic_topography)^11,13^. Sea ice concentration was taken from the NASA Bootstrap data archived by NSIDC^43^ and available via https://nsidc.org/data/nsidc-0079/. Beaufort Gyre Exploration Project CTD surveys are available via https://www.whoi.edu/page.do?pid=66521/. Derived time series presented in Fig. 2 are available as Source Data.

## Source Data

Source Data

## Code availability

The code generated during this study is available from the corresponding author upon reasonable request.
