# Supplementary material for: Enhanced eddy activity in the Beaufort Gyre in response to sea ice loss
Source: Nat Commun. 2020 Feb 6;11:761. doi: 10.1038/s41467-020-14449-z (PMC7005044; doi:10.1038/s41467-020-14449-z)
Supplement: Supplementary file 2 — Supplementary Information [file 41467_2020_14449_MOESM2_ESM.pdf]

Supplementary Information for "Enhanced eddy activity in the Beaufort Gyre in response to sea ice loss"

Thomas W. K. Armitage[1], Georgy E. Manucharyan[2], Alek A. Petty[3,4], Ron Kwok[1], Andrew F. Thompson[5]

[1]Jet Propulsion Laboratory, California Institute of Technology, Pasadena, California, USA.

[2]School of Oceanography, University of Washington, Seattle, WA.

[3]Cryospheric Sciences Laboratory, NASA Goddard Space Flight Center, Greenbelt, Maryland, USA

[4]Earth System Science Interdisciplinary Center, University of Maryland, College Park, Maryland, USA

[5]Environmental Science and Engineering, California Institute of Technology, Pasadena, California, USA.br

Corresponding author: T. W. K. Armitage (Tom.W.Armitage@jpl.nasa.gov)

Source Data

The derived time series in Figure 2 are available in Source Data which is provided along with the main manuscript.

Supplementary Figures

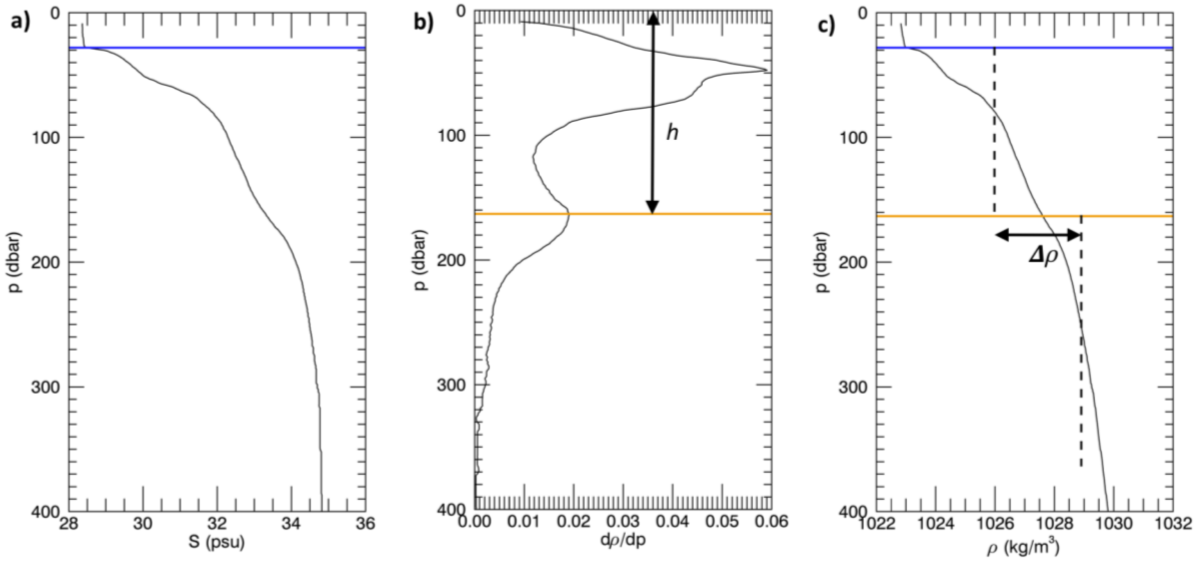

Supplementary Figure 1. Example salinity **(a)**, rate of change of density with depth **(b)**, and density **(c)** profiles recorded by a CTD in the Beaufort Gyre region. The horizontal lines represent the mixed layer depth (blue) and halocline depth, *h* (orange), picked out by the methodology described in the text. The vertical dashed lines in **(c)** represent the mean density in the halocline layer and immediately below.

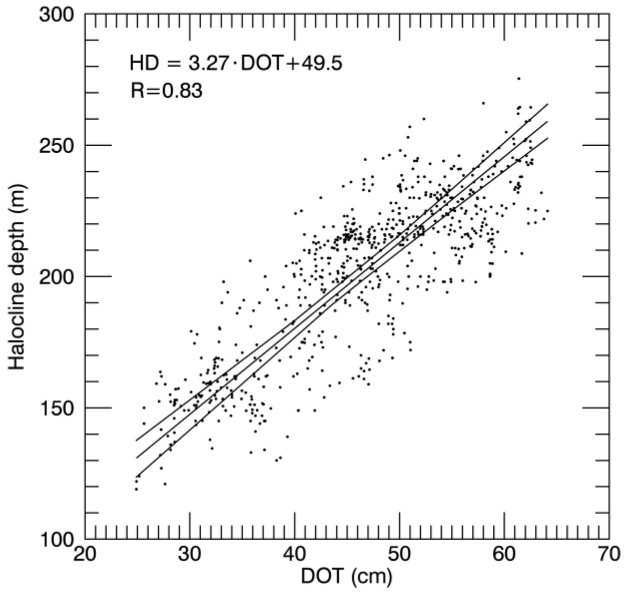

Supplementary Figure 2. Scatter plots of halocline depth estimated from BGEP mooring and CTD data against the concurrent dynamic ocean topography (DOT).

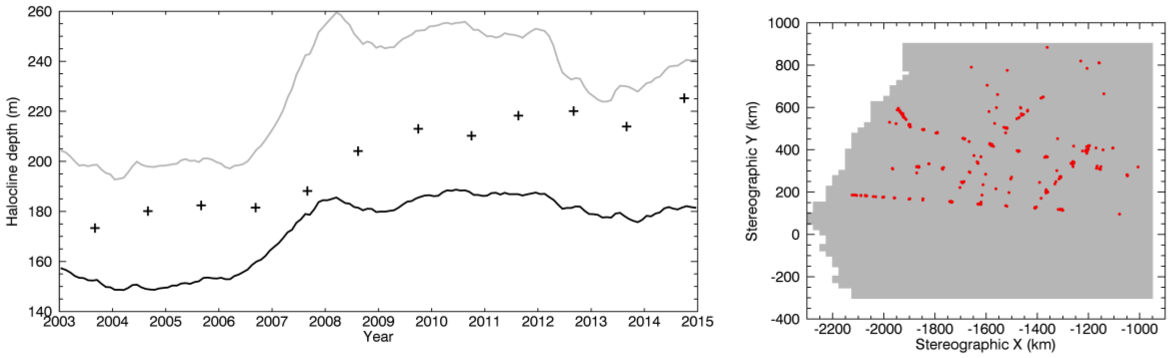

Supplementary Figure 3. Left: The mean halocline depth in the Beaufort Gyre region (thick line, m) and the center of the BG region (thin gray line, m) from the dynamic ocean topography data, and from BGEP CTD surveys (crosses). Right: The CTD station locations (red) overlain on the BG averaging region (gray).

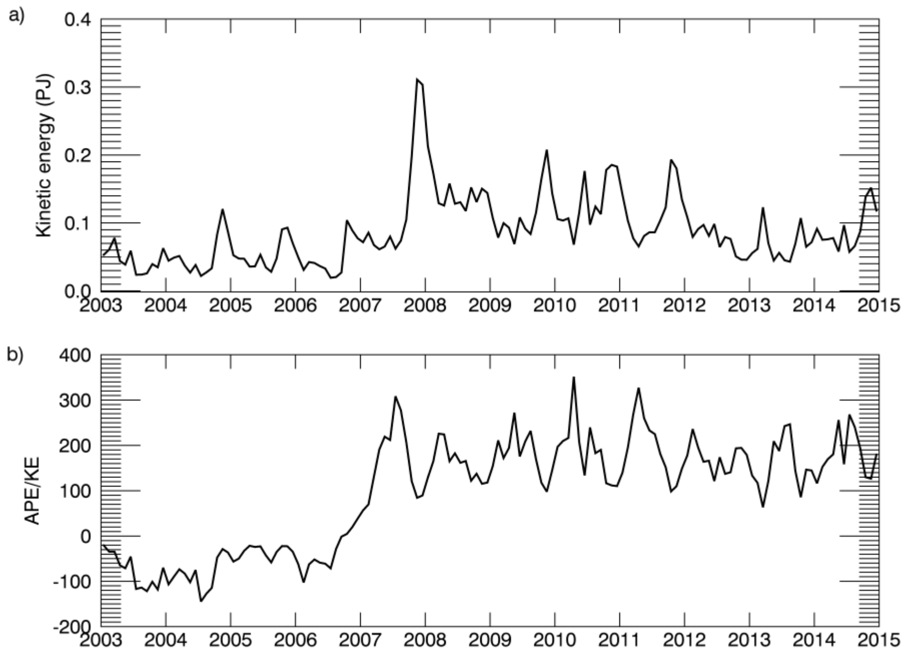

Supplementary Figure 4. The kinetic energy in the Beaufort Gyre region **(a)** and the ratio of available potential energy to kinetic energy **(b)**.
