# Supplementary material for: Enhanced eddy activity in the Beaufort Gyre in response to sea ice loss
Source: Nat Commun. 2020 Feb 6;11:761. doi: 10.1038/s41467-020-14449-z (PMC7005044; doi:10.1038/s41467-020-14449-z)
Supplement: Supplementary file 1 — Peer Review File [file 41467_2020_14449_MOESM1_ESM.pdf]

Reviewers' comments:

Reviewer #1 (Remarks to the Author):

The manuscript “Enhanced eddy activity in the Beaufort Gyre (BG) in response to sea ice loss” by Armitage et al. presents results of calculations and analysis of kinetic and available potential energy in the Beaufort Gyre region speculating that equilibration of the BG dynamics is a result of increased eddy activity. I recommend this paper for publication after substantial major revision.

This paper as an attempt to support BG-related theoretical and numerical conclusions of Manucharyan and Spall (2016) and Manucharyan et al., 2016, 2017) employing simple calculations of energy balance based on available and more or less accurate daily products (wind, ice concentration, ice drift) and monthly dynamic ocean topography.

After reading this manuscript, I cannot formulate specific outstanding features of this work. First, it is well known that oceanic kinetic energy is dominated by eddies, on average by a factor of 150. I think, it would be outstanding to show/calculate kinetic energy of eddies in the BG region. Some useful information about this is available in Zhao et al, 2015, 2016, 2018 but it is not used in this paper. In the paper, I found only speculation that reduction of APE is mainly associated with generation of eddies. On the other hand, the kinetic energy of eddies is not estimated and processes of eddy dissipation and transport of water properties in the BG are not well understood and studied. Secondly, all methods/algorithms of calculations were recently published and many results discussed (Giles et al., 2012; Dewey et al., 2018; Meneghello et al., 2017, 2018; Zhong et al., 2018). This is well outlined in this manuscript.

I think that one of the problems of this paper is that the BG region is considered as a closed system (equations 1 and 2) where fluxes of energy via boundaries are not taken into account. In the revised manuscript is would be important to justify this approach or/and estimate changes of the BG region APE and KE due exchanges with surrounding regions.

The second problem is that the calculations of halocline depth used for estimation of available potential energy are very uncertain. This is a weakest part of this work. From Figure S5 I can conclude that halocline depth after 2009 is stable or increases (at the same time FWC is also more or less stable except 2012 which correlates well with wind forcing) but in figure 2c it is shown that APE has a negative trend. This could be related to the water density gradient but it changes are not discussed.

Some other minor questions:

It is not clear how kinetics energy dissipation/input in the southern BG can influence freshwater accumulation (available potential energy) in the BG center and its stabilization.

Lines 37-38: This can be also due to absence of fresh water. In a barotropic ocean no fresh water accumulation will be observed.

Note that in the future, the increased mixing in the closed BG system has to reduce gradients of and result in the reduction of eddy activity, I think.

References

Manucharyan G.E., A.F. Thompson, and M.A. Spall (2017), Eddy-Memory mode of multi-decadal variability in residual-mean ocean circulations with application to the Beaufort Gyre, Journal of Physical Oceanography, 47, 855-866.

Manucharyan G.E., M.A. Spall, and A.F. Thompson (2016), A theory of the wind-driven Beaufort

Gyre variability, Journal of Physical Oceanography, 46, 3263-3278.

Manucharyan G.E. & M.A. Spall (2016), Wind-driven freshwater buildup and release in the Beaufort Gyre constrained by mesoscale eddies, Geophysical Research Letters, 43(1), pp 273-282.
Zhao, M., and M.-L. Timm e rm a n s (201 5) , Vertical scales and dynam i cs of eddies in the Arctic O cea n ’ s Canada Basin, J. Geophys. Res., 120 (12), 819 5– 82 0 9, doi :10.1002/2015JC01125 1.

Zhao, M., M.-L. Timmermans, S. Cole, R. Krishfield, A. Proshutinsky, and J. Toole (2014), Characterizing the eddy field in the Arctic Ocean halocline, J. Geophys. Res., 119 (12), 8800–8817, doi: 10.1002/2014JC00488.

Zhao, M., M.-L. Timmermans, S. Col e, R. Kr ishfield , and J. Toole (2016), Evolution of the eddy field in the Arctic Ocean’s Canada Basin , 2005–2015, Geophys. Res. Lett., 43 (15), 8106–8114, doi :10.1002/2016G0069671.

Zhao M., M.-L. Timmermans, R.A. Krishfield, and G.E Manucharyan. (2018), Partitioning of Kinetic Energy in the Arctic Ocean's Beaufort Gyre, J. Geophys. Res.Oceans. https://doi.org/10.1029/2018JC014037

Reviewer #2 (Remarks to the Author):

By computing an energy budget over the Beaufort Gyre, the authors provide an estimate of the baroclinic eddies's role in the equilibration of the Beaufort Gyre under changing ice-cover condition. This is a central problem in our understanding of the Arctic freshwater content equilibration and Beaufort Gyre circulation, and I recommend publication provided the points outlined below are addressed.

1) at the monthly time scale, sea surface variability is much larger than isopycnal depth variability. As a consequence, using the monthly measured sea surface height to infer h in equation (3) potentially results in an overestimate of APE. I would expect equation (5) to be valid only for longer time scales. Otherwise said, the isopycnal depth shown in Figure S5 is probably closer to a linear interpolation of the crosses rather the highly variable black and gray lines.

2) I do not fully understand the concept of "KE energy input" (e.g. line 152 and elsewhere in the manuscript). I would expect a wind/ice driven energy input to be redistributed to KE and APE according to the system dynamics. In the hp of gesotrophic flow with zero bottom pressure gradient, which I understand are at the basis of this study, APE and KE are strictly related because ocean velocity is only a diagnostic variable. Could the authors clarify this point?

3) ice in free-drift is not mentioned in the paper, but it has an important role in changing the momentum transfer between the atmosphere and the ocean. A gyre with free drifting ice will require even more energetic eddies to equilibrate than an ice-free gyre. Following this argument, wouldn't the sign of Ekman pumping in the ice covered region depend on the ice concentration and thickness?

4) Some notation issues:
- equation 1 should have a total derivative in place of a partial derivative
- equation 3 integrates a constant, given that \bar{h^2} and \bar{h}^2 are already averaged over the BG. It would then be enough to multiply by the area
- equation 4: should u and v depend on the horizontal position (lat,lon) too?
- line 335: the "ekman layer velocity" should be the "ekman layer transport velocity" to better differentiate from the surface ekman velocity above

- Figure S4: I understand that least square is used to infer the relationship between dynamic ocean topography and halocline depth, but both have associated uncertainties so Orthogonal distance regression would be more appropriate. This could affect the estimate of APE (in addition to what said on point 1).

I hope the authors will find my comments useful.

Best

Gianluca Meneghello

Reviewer #1

The manuscript "Enhanced eddy activity in the Beaufort Gyre (BG) in response to sea ice loss" by Armitage et al. presents results of calculations and analysis of kinetic and available potential energy in the Beaufort Gyre region speculating that equilibration of the BG dynamics is a result of increased eddy activity. I recommend this paper for publication after substantial major revision.

> We thank the reviewer for their constructive and thoughtful comments. We have addressed each point below and we feel that the manuscript is greatly improved as a result.

This paper as an attempt to support BG-related theoretical and numerical conclusions of Manucharyan and Spall (2016) and Manucharyan et al., 2016, 2017) employing simple calculations of energy balance based on available and more or less accurate daily products (wind, ice concentration, ice drift) and monthly dynamic ocean topography.

After reading this manuscript, I cannot formulate specific outstanding features of this work. First, it is well known that oceanic kinetic energy is dominated by eddies, on average by a factor of 150. I think, it would be outstanding to show/calculate kinetic energy of eddies in the BG region. Some useful information about this is available in Zhao et al, 2015, 2016, 2018 but it is not used in this paper. In the paper, I found only speculation that reduction of APE is mainly associated with generation of eddies. On the other hand, the kinetic energy of eddies is not estimated and processes of eddy dissipation and transport of water properties in the BG are not well understood and studied. Secondly, all methods/algorithms of calculations were recently published and many results discussed (Giles et al., 2012; Dewey et al., 2018; Meneghello et al., 2017, 2018; Zhong et al., 2018). This is well outlined in this manuscript.

To address your points:

1. In this study, we did not set out to *directly* calculate the KE of eddies, as you mention. There is some useful information on KE of eddies in the literature as you point out, which we cite, however we are not currently at a point where it is possible to calculate the gyre-scale KE of eddies directly due to limitations of the observational data: i) a lack of coverage from in situ instruments, and ii) a lack of resolution from satellites (horizontal resolution O(80km) vs. radius of deformation of 10-15 km in the Canada Basin). In the absence of this, we have attempted to infer information about the dissipative role of eddies by considering the mechanical energy budget – the dissipation of energy by eddies is directly related to their KE, and in this way our calculation is an indirect bulk measurement of eddy KE.
2. We would assert that the 'outstanding feature' of the work is not revealing new physics/dynamics of the of the BG system, but for the first time quantifying the gyre-scale energy budget using an observational approach, how it has changed in recent years, and the implications of the changes for the future. Estimating the role of eddies and ice/wind energy sources and sinks for the BG is a novel approach (no papers are currently published on this topic) and one of considerable

interest given recent modelling studies about the role of eddies and the ice-ocean governor in the BG. With respect to the theoretical and modeling communities, much of the work eddies in the Arctic have been based on idealized modeling efforts or model that require parameterization for boundary layer processes. Thus, confirming, with observation, theories that have grown out of this work is a key step in advancing our understanding of the Arctic Ocean.

3. While we have not presented any new primary data here, and some of the derived data has been presented elsewhere (and are properly referenced), it is not true that "all methods/algorithms of calculations were recently published". In particular, our treatment of the *in situ* data and subsequent comparison against the DOT is novel, as is the estimation of halocline depth, APE, wind work, and KE of the mean geostrophic flow from satellite altimetry data in the Arctic. Further, as we note above, the key novelty is this is the first attempt to characterize the changing state of the BG system by considering a closed mechanical energy budget, a method that has not been applied in the Arctic before. This allows us to infer information about the role of eddies at a bulk scale, which is currently unfeasible using the available observations.

I think that one of the problems of this paper is that the BG region is considered as a closed system (equations 1 and 2) where fluxes of energy via boundaries are not taken into account. In the revised manuscript is would be important to justify this approach or/and estimate changes of the BG region APE and KE due exchanges with surrounding regions.

Thank you for this important comment. To address the energy flow through the gyre boundaries, we have reformulated the APE evolution equation in a more rigorous way that accounts for boundary thickness fluxes due to Ekman transport (see Methods and Supplementary Materials). This term is important but has a small effect compared to the other terms (see pink line in Fig 2a). Accounting for the boundary terms in our energy budgets changes slightly our estimate for the eddy dissipation term, but does not affect the key conclusions of the paper.

The second problem is that the calculations of halocline depth used for estimation of available potential energy are very uncertain. This is a weakest part of this work. From Figure S5 I can conclude that halocline depth after 2009 is stable or increases (at the same time FWC is also more or less stable except 2012 which correlates well with wind forcing) but in figure 2c it is shown that APE has a negative trend. This could be related to the water density gradient but it changes are not discussed.

Both yourself and the other reviewer picked up on this point, and we have attempted to improve our treatment of the Hd estimation in this revision. First, in response to the other reviewer, we are now estimating Hd from the 12-month-smoothed DOT data. This improves the correlation with the CTD-derived Hd as well as the regression between the DOT and CTD-derived Hd – we refer you to the discussion below in response to the other reviewer for further details and plots. As well as this, the other reviewer

recommended an 'orthogonal distance regression' rather than a simple linear least squares regression (see below), which also improves the regression.

In the revised manuscript we have also taken the uncertainty in the DOT/Hd regression to estimate a range of uncertainty on the APE calculation, which also affects the range of estimated uncertainty on the eddy dissipation term (see updated figure 2). After incorporating this into our calculations we find the uncertainty range is smaller than the interannual change in APE and eddy dissipation that we see after 2007.

Some other minor questions:

It is not clear how kinetics energy dissipation/input in the southern BG can influence freshwater accumulation (available potential energy) in the BG center and its stabilization.

This is a great comment: identifying pathways of energy propagation from its sources to sinks remains a challenge for theoretical oceanography. We can only speculate that the energy propagation occurs via preferential mesoscale eddy propagation away from the southern BG region (where the strong baroclinic currents near gyre boundaries facilitate eddy formation) towards the interior of the gyre where there is mechanical dissipation; as the eddies propagate to the interior of the gyre, they carry not only the freshwater anomalies (halocline thickness anomalies) but also the eddy available potential energy and eddy kinetic energy. We make note of this in our Discussion section.

Lines 37-38: This can be also due to absence of fresh water. In a barotropic ocean no fresh water accumulation will be observed.

It is true that in a closed system without freshwater sources and sinks (e.g. in a barotropic ocean) the FWC must be conserved. Nonetheless, hydrographic observations of the BG show strong baroclinicity and increasing FWC over recent decades.

Note that in the future, the increased mixing in the closed BG system has to reduce gradients of and result in the reduction of eddy activity, I think.

We agree that the eddy activity is indeed counteracting the formation of mean currents and, in the absence of forcing, eddies would eventually drain the entire energy from the gyre and dissipate themselves. However, the gyre is a forced system and the eddy activity depends on a balance between forcing and dissipation. The observations suggest that with the reduction of sea ice the gyre receives a larger amount of mechanical energy and hence the eddy activity is expected to increase as well.

References

Manucharyan G.E., A.F. Thompson, and M.A. Spall (2017), Eddy-Memory mode of multi-decadal variability in residual-mean ocean circulations with application to the Beaufort Gyre, Journal of Physical Oceanography, 47, 855-866.

Manucharyan G.E., M.A. Spall, and A.F. Thompson (2016), A theory of the wind-driven Beaufort Gyre variability, Journal of Physical Oceanography, 46, 3263-3278.

Manucharyan G.E. & M.A. Spall (2016), Wind-driven freshwater buildup and release in the Beaufort Gyre constrained by mesoscale eddies, Geophysical Research Letters, 43(1), pp 273-282.

Zhao, M., and M.-L. Timm e rm a n s (201 5) , Vertical scales and dynam i cs of eddies in the Arctic O cea n ' s Canada Basin, J. Geophys. Res., 120 (12), 819 5– 82 0 9, doi :10.1002/2015JC01125 1.

Zhao, M., M.-L. Timmermans, S. Cole, R. Krishfield, A. Proshutinsky, and J. Toole (2014), Characterizing the eddy field in the Arctic Ocean halocline, J. Geophys. Res., 119 (12), 8800–8817, doi: 10.1002/2014JC00488.

Zhao, M., M.-L. Timmermans, S. Col e, R. Kr ishfield , and J. Toole (2016), Evolution of the eddy field in the Arctic Ocean's Canada Basin , 2005–2015, Geophys. Res. Lett., 43 (15), 8106–8114, doi :10.1002/2016G0069671.

Zhao M., M.-L. Timmermans, R.A. Krishfield, and G.E Manucharyan. (2018), Partitioning of Kinetic Energy in the Arctic Ocean's Beaufort Gyre, J. Geophys. Res.Oceans. https://doi.org/10.1029/2018JC014037

Reviewer #2

By computing an energy budget over the Beaufort Gyre, the authors provide an estimate of the baroclinic eddies's role in the equilibration of the Beaufort Gyre under changing ice-cover condition. This is a central problem in our understanding of the Arctic freshwater content equilibration and Beaufort Gyre circulation, and I recommend publication provided the points outlined below are addressed.

> Gianluca – thanks for your interest and constructive feedback on the manuscript. We have addressed your comments below and feel that the work is greatly improved as a result of your input.

1) at the monthly time scale, sea surface variability is much larger than isopycnal depth variability. As a consequence, using the monthly measured sea surface height to infer h in equation (3) potentially results in an overestimate of APE. I would expect equation (5) to be valid only for longer time scales. Otherwise said, the isopycnal depth shown in Figure S5 is probably closer to a linear interpolation of the crosses rather the highly variable black and gray lines.

> We have compared the DOT and CTD-derived halocline depth and re-calculated the APE using the time-smoothed DOT, for smoothing windows of 12, 24, and 36 months.

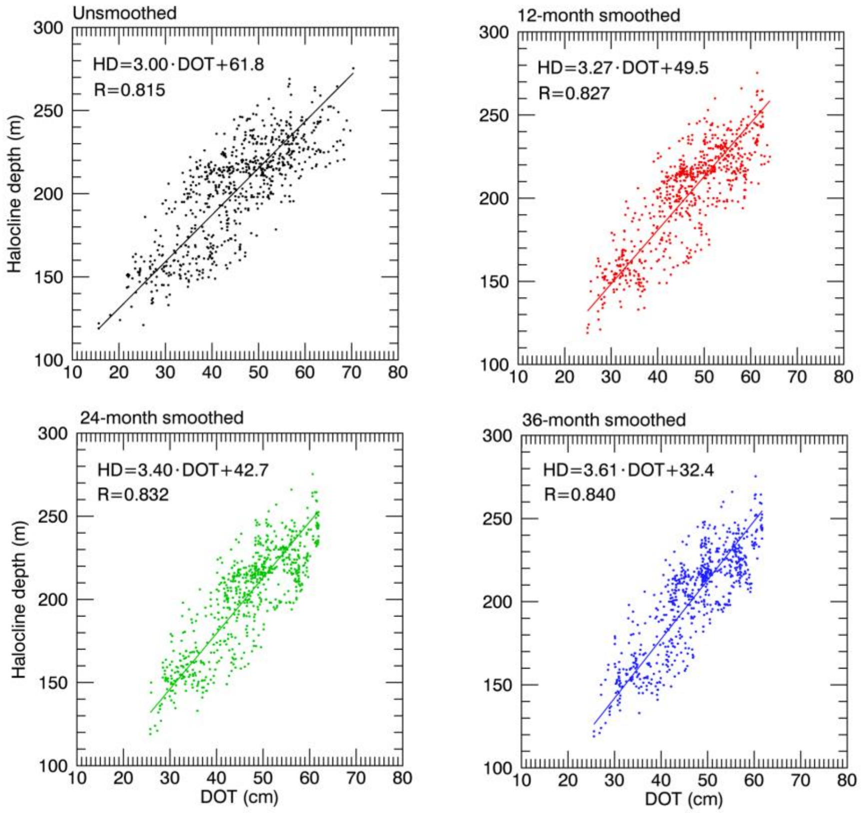

This figure shows the halocline depth derived from BGEP CTDs against the spatially and temporally coincident DOT, their correlation, and best fit for different time-smoothing of the DOT data (i.e., similar to Figure S4 in our original manuscript). As you can see, the amount of smoothing increases the correlation and changes the line of best fit.

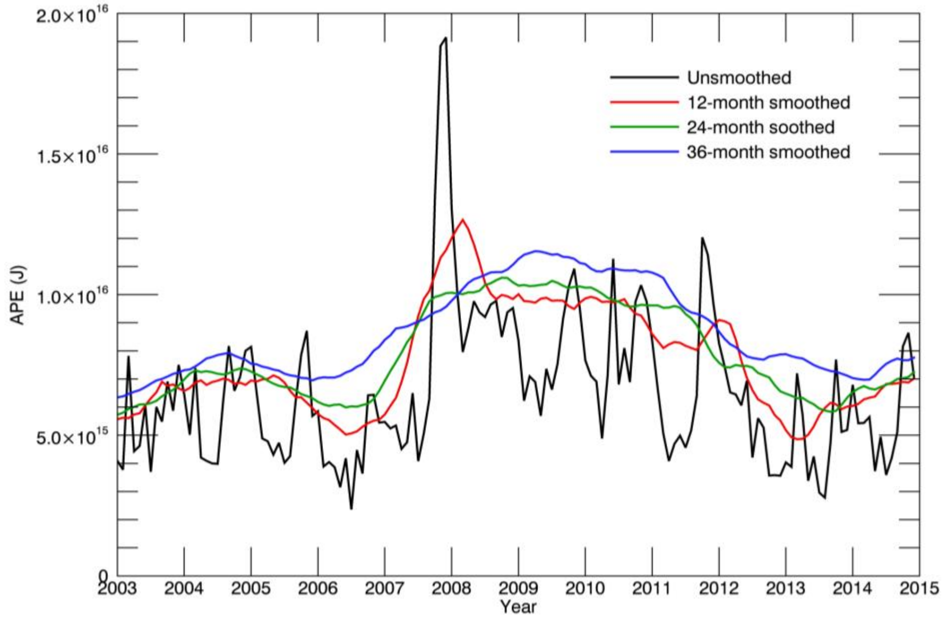

This figure shows the resulting APE calculation using the 4 different regressions show in the above figure. So, while the amount of DOT time-smoothing does affect the resulting APE time series, the magnitude is similar and the variability on interannual time scales is also similar. We do agree that the isopycnal depth variability is smaller than the DOT variability, and hence in our revised manuscript we used the 12-month smoothed DOT in the Hd regression and to calculate the APE. This is a compromise between improved regression and not introducing too large “edge” effects at the beginning and end of the time series, or smoothing out the signal, as well as the fact that using longer time-smoothing does not qualitatively or quantitatively change our results/conclusions (see the APE time series comparison above).

2) I do not fully understand the concept of "KE energy input" (e.g. line 152 and elsewhere in the manuscript). I would expect a wind/ice driven energy input to be redistributed to KE and APE according to the system dynamics. In the hp of gesotrophic flow with zero bottom pressure gradient, which I understand are at the basis of this study, APE and KE are strictly related because ocean velocity is only a diagnostic variable. Could the authors clarify this point?

When we were referring to “KE energy input” we were simply referring to the work done by the ocean surface stress on the geostrophic currents, i.e., $W = \tau \cdot u_g$, the wind power input. Note that this term appears most directly in the KE evolution equation as a result of the vertical stress divergence in the momentum equation. It appears, slightly less intuitively, in the APE evolution equation through the correlation of Hd with the wind stress curl and the fact that $u_g$is related to the gradient of Hd. We understand why this may have been confusing, you are correct that the wind stress work will be redistributed

throughout the system as KE and APE – of course, the BG is a strongly baroclinic flow, meaning the majority of wind stress energy input goes into increased APE. We have clarified this in the paper and we have changed "KE energy input" to "wind energy input" in order to make it clear what we a referring to.

3) ice in free-drift is not mentioned in the paper, but it has an important role in changing the momentum transfer between the atmosphere and the ocean. A gyre with free drifting ice will require even more energetic eddies to equilibrate than an ice-free gyre. Following this argument, wouldn't the sign of Ekman pumping in the ice covered region depend on the ice concentration and thickness?

You are correct that an Arctic where sea ice is free-drifting for larger portions of the year will require more energetic eddies to dissipate the energy. As you have pointed out in your recent work, under free drift conditions, the negative feedback between ice-ocean stress and surface currents (the governor effect) switches off in free drift conditions, which will affect the magnitude/sign of Ekman pumping, and in turn whether or not ice is free-drifting depends on its concentration and thickness.

We note however that in our calculations free-drifting ice is implicitly considered because the sea ice motion data products that we use capture ice motion when it is both consolidated (and not free-drifting) as well as unconsolidated (and free-drifting).

4) Some notation issues:

- equation 1 should have a total derivative in place of a partial derivative

Changed in the revision.

- equation 3 integrates a constant, given that \bar{h^2} and \bar{h}^2 are already averaged over the BG. It would then be enough to multiply by the area

Changed in the revision.

- equation 4: should u and v depend on the horizontal position (lat,lon) too?

Changed in the revision.

- line 335: the "ekman layer velocity" should be the "ekman layer transport velocity" to better differentiate from the surface ekman velocity above

Changed in the revision.

- Figure S4: I understand that least square is used to infer the relationship between dynamic ocean topography and halocline depth, but both have associated uncertainties so Orthogonal distance regression would be more appropriate. This could affect the estimate of APE (in addition to what said on point 1).

This is good point - in the revision we have used an orthogonal distance regression and it does affect the fit (see plots above in response to your first point and in the updated manuscript).

Reviewers' comments:

Reviewer #2 (Remarks to the Author):

Review for "Enhanced eddy activity in the Beaufort Gyre in response to sea ice loss" By T. Armitage et al.

By closing an observationally-constrained energy budget, the paper attempts to show that eddy activity in the Beaufort Gyre has increased after 2007.

I thank the authors for trying to address my comments, and I am very happy if I could have been of help. I am still convinced that the topic discussed in the paper is interesting and worth publication, and that the conclusions might be correct. Even the simplest eddy diffusivity closure would suggest that an increase in isopycnal depth anomaly would result in an increase in eddy fluxes: isopycnal depth anomaly has increased by ~50% in 2007, and we can expect eddy fluxes to have increased accordingly. Indeed, the very same conclusions --- outlined on line 40-43 of the abstract --- have been already suggested in two papers from my colleagues and myself: Meneghello et al., 2018a and Doddridge et al., 2019. Based on the analysis of idealized analytical models, both conclude that less sea ice would result in a reduced efficiency of the ice-ocean governor and an increase in eddy activity. Doddridge et al., 2019 in particular discusses the relative role of the governor and eddy fluxes in balancing the gyre. Observational evidence of an increase in eddy activity has been presented by Zhao et al. (2016), based on in-situ observations.

A more large scale approach would be very welcome and I am convinced that this work would be an interesting addition to the current literature. At the same time, it is my opinion that the way results are presented still leaves some questions unanswered. Further questions have arised with the advancement of the topic since my last review. I will list them below.

1) The conclusions of this work, that eddy activity has increased starting from 2007, rests on two assumptions: a) that the Beaufort Gyre's isopycnal depth anomaly (and hence potential energy) can be inferred from observations of sea surface height with sufficient accuracy and that b) all variations in potential energy not taken into account by wind work and Ekman transport are due to eddies. These assumptions are summarized by equation #4.

a) Concerning the first assumption, I suggested in my last review that isopycnal depth cannot be directly inferred from sea surface height: the former responds to wind/ice forcing on a much longer time scale that the latter. The authors addressed my comment by computing a 12 months running mean. While I do appreciate that correlation of sea surface height with the observed isopycnal depth anomaly increases after smoothing, the smoothing operation introduces a paradox: the computed isopycnal depth at any time depends on the value of sea surface height six months in the future. As the authors themselves admit, this "means that the estimated halocline depth is likely inaccurate during period of rapid change" (line 387-388). I would add that such estimate is likely inaccurate during any period of change, and that a running mean is not an appropriate filter.

b) Concerning the second assumption, I have two comments. First, only part of the wind/ice work computed by the authors is actually acting on the isopycnal depth. This is discussed, e.g., in Roquet et al. (2011), showing how the power input on the isopycnal is proportional to the product of the vertical pressure gradient and the Ekman pumping, and that this is only part of tau_0*u_g. Second, and possibly more importantly, it seems to me there is a mismatch between the daily time scales over which energy input is computed, and the much longer time scales used for estimating energy dissipation. Specifically, the authors neglect bottom dissipation based on the fact that "the western Arctic is highly baroclinic, and currents at depth are an order of magnitude weaker than at the surface" (line 190-191). This is certainly true at long time scales but at short time scales a barotropic flow, dissipating energy at the bottom of the ocean, is present and should

be discussed (or removed from the energy input if that is possible).

Given that these are the two assumptions on which all conclusions are based, I would ask the authors to be clearer on these two points.

2) the argument in favor of an increase in eddy activity, as outlined in the abstract, should be made clearer. The authors state (line 36-40) that

"The dramatic loss of sea ice and acceleration of surface currents after 2007 led to a net annual wind energy input to the BG, while the potential energy stored in the BG halocline decreased. Meanwhile, FWC remained stable despite net Ekman downwelling. To balance this, energy dissipation and FWC equilibration by oceanic eddies must also have increased relative to the ice-ocean governor mechanism after 2007."

In the geostrophic, baroclinically adjusted limit used by the authors surface currents are a diagnostic variable of potential energy so that if one increases the other one has to increase too (and viceversa). Within the assumption of constant density difference used by the authors, the same should hold for FWC too. This can be deduced by equation #1 and #3 of the paper itself: on time scales longer than a year or so, both currents and FWC are a function of the isopycnal depth anomaly. Given that the decrease in potential energy is used as a the main proxy for an increase in eddy activity, what is increasing and what is decreasing should be made clearer. Incidentally, the geostrophic constrain is also the reason why kinetic energy is always two order of magnitudes smaller than potential energy, as stated in the section starting on line 390 (see also line 117-118).

3) Some additional clarification about how the energy budget is closed would be very useful. I have not tried to close it myself, but a freshwater budget should give comparable results, especially in light of the discussion of the proportionality between Ekman pumping and power input above. In addition to the freshwater comment in the abstract, the author states that (line 63-65, see also line 86-90)

"However, the time-mean area-averaged Ekman pumping across the BG is persistently negative, implying a tendency for halocline deepening and freshwater accumulation that cannot continue in perpetuity and must ultimately be balanced by other processes."

According to my own estimates (see http://mgl.mit.edu/pdf/meneghello2018exploring.pdf, currently under review), focusing on the same period studied by the authors, the mean 2003-2014 Ekman downwelling can be accounted as follows:

- 12.2 m/y of ice and wind driven downwelling (neglecting the ice-ocean feedback)
- 9.8 m/y of ice-ocean feedback driven "upwelling"

The residual 2.4m/y downwelling is taken into account by

- 1.8 m/y of eddy driven "upwelling"
- 0.6 m/y downwelling accumulated in the 7m isopycnal depth increase over 12 years.

Model- and observation-based estimates by Zhong et al. (2018) reports similar values for the residual Ekman pumping. In either case, the freshwater budget is closed as all freshwater accumulated by Ekman convergence is accounted for in the isopycnal depth increase.

This does not mean that eddy activity has not increased: as pointed out above, even the simplest eddy diffusivity closure suggests an increase in eddy flux of ~50% as a consequence of the 7m, or ~50%, increase in isopycnal depth anomaly, but this is not the argument used by the authors. Their conclusions seems to be based on the numbers presented in the paragraph starting on line 158: for 2008-2014 "there was net wind energy input of ~ 2.1 PJ/year, an APE decline of 1.7

PJ/year and a dissipation by eddies of 4.3 PJ/year". Provided the wind energy input is a good estimate (see discussion about the second assumption above), this leaves 0.5 PJ/year of energy input unaccounted for. Large scale kinetic energy is not a candidate because it is negligible. From eq. 4 I suppose the missing term is Ekman boundary flux, but then the sign is wrong. The cumulative energy associated to ekman boundary flux seems to decrease in Figure 2a, implying an energy output, not an input. I tried to ball park the numbers from Figure 2a and 2c, between 2009 and 2014 we have

- 40 PJ added by atmospheric ocean stress work
- 30 PJ removed by ice-ocean stress work
- ~1 PJ removed by ekman boundary flux
- ~2 PJ of potential energy loss

leaving ~7 PJ to be dissipated by eddies to close the balance. These numbers are consistent with the 2003-2014 Ekman pumping balance discussed before: in both the Ekman pumping and the energy budget about 75-80% of the input is taken into account by the ice, and the 14-17% by the eddies. That would correspond to an eddy dissipation of 1 PJ/y rather than the 4.3 PJ/year suggested by the authors. Mine is clearly not a rigorous analysis, but I hope it clarifies where I am confused.

4) Another claim made by the authors is that eddies are required to transfer energy from the southern region, when it enters the gyre, to the northern part, where it is dissipated. A much simpler mechanism doing that is the geostrophic current itself: energy will indeed enter in the ice-free south and be dissipated in the ice-covered north, but it would be transported by the pressure field driving the current.

5) in the paragraph starting on line 243, the authors write:

"Dissipation of energy underneath sea ice will also increase as currents speed up, however, as sea ice continues to decline for longer periods of the year, its cumulative capacity to dissipate upper open energy will diminish".

Energy dissipation varies linearly with the ice cover, but with the cube of the current speed, as pointed by the author themselves on one 177. This conclusions is not as obvious as it seems.

In conclusion, while I do agree with the idea that i) eddies are critical processes fo the accurate representation of the BG systems in models, ii) that eddy activity has probably increased after 2007, and iii) that closing the energy budget has indeed the potential to show it, I suggest a more detailed is necessary to show that eddy activity "must have increased".

Other minor comments below:

line 114: no model or layers have been mentioned before this point

line 151-152: The author states that "However the eddy response may lag the forcing by a few years [Manucharyan et al., 2017].". But Manucharyan et al. 2017 studies the response of the entire Beaufort Gyre, not the response of the eddy themselves. The eddies respond to a change of the isopycnal slope on the time scale of baroclinic instability, which should be a couple of weeks.

line 236-242: the ice-ocean governor is not needed to balance the Ekman pumping if the Ekman pumping itself is upwelling instead of downwelling. The same is true for the eddies.

Figures: many panels show work with units of Watts. Power is measured in Watts, work in Joules.

Equation 6: Ai is not defined

line 333: the values of Cdao and Cdio used should be made explicit

line 500: this citation is repeated (and some dois seems wrong in many other citations)

======================

Bibliography

E. W. Doddridge, G. Meneghello, J. Marshall, J. Scott, C. Lique (2019) A Three-way Balance in The Beaufort Gyre: The Ice-Ocean Governor, Wind Stress, and Eddy Diffusivity. J. Geophys. Res. C: Oceans, doi:10.1029/2018JC014897

G. Meneghello, J. Marshall, J.-M. Campin, E. W. Doddridge, M.-L. Timmermans (2018a) The Ice-Ocean governor: ice-ocean stress feedback limits Beaufort Gyre spin up. Geophys. Res. Lett., 45. doi:10.1029/2018GL080171

Roquet, Wunsch and Madec (2011) On the Patterns of Wind-Power Input to the Ocean Circulation JPO https://doi.org/10.1175/JPO-D-11-024.1

Zhao, M., M.-L. Timmermans, S. Cole, R. Krishfield, and J. Toole (2016), Evolution of the eddy field in the Arctic Ocean's Canada Basin, 2005–2015, Geophys. Res. Lett., 43, 8106–8114, doi 10.1002/2016GL069671

Zhong, W., Steele, M., Zhang, J., & Zhao, J. (2018). Greater role of geostrophic currents in Ekman dynamics in the western Arctic Ocean as a mechanism for Beaufort Gyre Stabilization. Journal of Geophysical Research: Oceans, 123, 149–165. https://doi.org/10.1002/2017JC013282

Additional comments on "Enhanced eddy activity in the Beaufort Gyre in response to sea ice loss" by T. Armitage et al.

In addition to my already submitted review, I was asked by the editor to comment on the concerns of reviewer 1.

While I agree with reviewer 1 that large part of the methods used have been previously discussed, and that similar conclusions has been published in recent work, large-scale observational evidence of the evolution of the eddy field in the Arctic is indeed missing and will be very welcome by me and, I think, by the Arctic Oceanography community in general. I am convinced that the topic addressed is very current, and that a paper covering it should be considered for publication.

At the same time, I partly agree with reviewer 1 when he states that "I found only speculation that reduction of APE is mainly associated with generation of eddies". This might be too strong a statement: closing the energy budget is a very welcome attempt to ground such speculation in observations. What remains speculative is the idea that the terms considered in the energy budget cover everything: other components of the energy budget are quickly dismissed by the authors with very little discussion. An example is energy dissipation by bottom flow, see discussion in my last review. These might be indeed negligible on long time scales --- corresponding to an equilibrated gyre --- but they are most probably not at the short time scales discussed here. More importantly there is a mismatch between the daily time scale at which the energy input is computed, and the long time scale at which the energy budget in its current form can be considered closed.

Concerning the estimation of APE, I agree with reviewer 1 that it is not clear how halocline depth and FWC are stable or increasing after 2007, but APE decreases. In the framework used by the authors, I would have expected the three to be closely related, and the text is not clear on why this is not the case (see discussion in my last review).

Summarizing, while I do think that the topic addressed by this study is of interest and deserves publication, and that closing an energy budget is an interesting approach to the problem, I also think a better justification and explanation of the hypothesis underlying the energy budget, or a modification of the budget itself, would be needed to address the concerns of reviewer 1 (and which in large part I share).

I hope my comments are helpful.

Reviewer #2

Review for "Enhanced eddy activity in the Beaufort Gyre in response to sea ice loss" By T. Armitage et al.

By closing an observationally-constrained energy budget, the paper attempts to show that eddy activity in the Beaufort Gyre has increased after 2007.

I thank the authors for trying to address my comments, and I am very happy if I could have been of help. I am still convinced that the topic discussed in the paper is interesting and worth publication, and that the conclusions might be correct. Even the simplest eddy diffusivity closure would suggest that an increase in isopycnal depth anomaly would result in an increase in eddy fluxes: isopycnal depth anomaly has increased by ~50% in 2007, and we can expect eddy fluxes to have increased accordingly. Indeed, the very same conclusions --- outlined on line 40-43 of the abstract --- have been already suggested in two papers from my colleagues and myself: Meneghello et al., 2018a and Doddridge et al., 2019. Based on the analysis of idealized analytical models, both conclude that less sea ice would result in a reduced efficiency of the ice-ocean governor and an increase in eddy activity. Doddridge et al., 2019 in particular discusses the relative role of the governor and eddy fluxes in balancing the gyre. Observational evidence of an increase in eddy activity has been presented by Zhao et al. (2016), based on in-situ observations.

A more large scale approach would be very welcome and I am convinced that this work would be an interesting addition to the current literature. At the same time, it is my opinion that the way results are presented still leaves some questions unanswered. Further questions have arised with the advancement of the topic since my last review. I will list them below.

> Thanks once more to the reviewer for providing helpful input on this manuscript, as well as for assessing our response to another reviewer. We appreciate that this has become a topic interest, and we are pleased that you agree that our treatment of the larger-scale energy budget of the Beaufort Gyre is an interesting addition to the field. We have addressed your concerns below.

1) The conclusions of this work, that eddy activity has increased starting from 2007, rests on two assumptions: a) that the Beaufort Gyre's isopycnal depth anomaly (and hence potential energy) can be inferred from observations of sea surface height with sufficient accuracy and that b) all variations in potential energy not taken into account by wind work and Ekman transport are due to eddies. These assumptions are summarized by equation #4.

a) Concerning the first assumption, I suggested in my last review that isopycnal depth cannot be directly inferred from sea surface height: the former responds to wind/ice forcing on a much longer time scale that the latter. The authors addressed my comment by computing a 12 months running mean. While I do appreciate that correlation of sea surface height with the observed isopycnal depth anomaly increases after smoothing, the smoothing operation introduces a paradox: the computed isopycnal depth at any time depends on the value of sea surface height six months in the future. As the authors themselves admit, this "means that the estimated halocline depth is likely inaccurate during period of rapid change" (line 387-388). I would add that such estimate is likely inaccurate during any period of change, and that a running mean is not an appropriate filter.

> We apply the smoothing as an attempt to filter out short-term processes that are unrelated to the long-term trends (e.g., month-to-month barotropic variability). Given the short observational record and coarse time resolution (monthly) of the ocean currents data, a simple running mean was the only straightforward was of addressing the filtering problem. Smoothing indeed improves the correlation between halocline depth and DOT, however it didn't affect the main results of the

paper, so this is not an essential methodological element on which the paper relies. While we agree that use of a 12-month smoothing introduces a paradox (which we now acknowledge explicitly in the text), we note that the main conclusions of our work depend on changes over multi-annual time periods rather than year-to-year changes i.e., over time periods longer than the 12-month smoothing that we apply. To reflect this in the revised manuscript we have focused our results on multi-annual changes rather than computing linear trends (see Figure 2 in the revised manuscript and related discussion, as well clarification in the Methods). These changes do not depend on the exact period of the running mean filter as long as the smoothing window is much shorter than the timescale of the dynamics in question.

b) Concerning the second assumption, I have two comments. First, only part of the wind/ice work computed by the authors is actually acting on the isopycnal depth. This is discussed, e.g., in Roquet et al. (2011), showing how the power input on the isopycnal is proportional to the product of the vertical pressure gradient and the Ekman pumping, and that this is only part of tau_0*u_g. Second, and possibly more importantly, it seems to me there is a mismatch between the daily time scales over which energy input is computed, and the much longer time scales used for estimating energy dissipation. Specifically, the authors neglect bottom dissipation based on the fact that "the western Arctic is highly baroclinic, and currents at depth are an order of magnitude weaker than at the surface" (line 190-191). This is certainly true at long time scales but at short time scales a barotropic flow, dissipating energy at the bottom of the ocean, is present and should be discussed (or removed from the energy input if that is possible).

On your first point: The main focus of our paper is to construct a domain-averaged view of the BG energy evolution. From this perspective our estimate of the energy input into geostrophic currents being equal to $\tau_o \cdot u_g$ is consistent with the idea presented in Roquet et al. (2011), i.e., it is equal to the product of the pressure gradient and Ekman pumping up to the divergence term that doesn't necessarily integrate to zero because of the open boundaries of the BG. However, Figure 1 shows the spatial distribution of the energy input, which could be affected if accounting only for the product of the pressure gradient and Ekman pumping instead of computing the full energy input $\tau_o \cdot u_g$. It is beyond the scope of our paper to discuss in more detail the exact processes of lateral energy distribution as we focus on area-integrated energy balance. We thus explicitly note in the text that the overall energy input $\tau_o \cdot u_g$ is partially redistributed laterally within the Ekman layer before entering the interior of the gyre and refer the reader to Roquet et al. (2011) paper for more information.

On your second point: See below a plot of the mean and standard deviation of $|u_g|^3$ as a function of depth from BGEP MMP data (mooring D). While there is a small mean flow and current variability at depth, it is 2—3 orders of magnitude smaller than at the surface, even at daily the time-scales captured by the mooring, and so we feel that we are justified to ignore the contribution of bottom drag relative to surface-based dissipation.

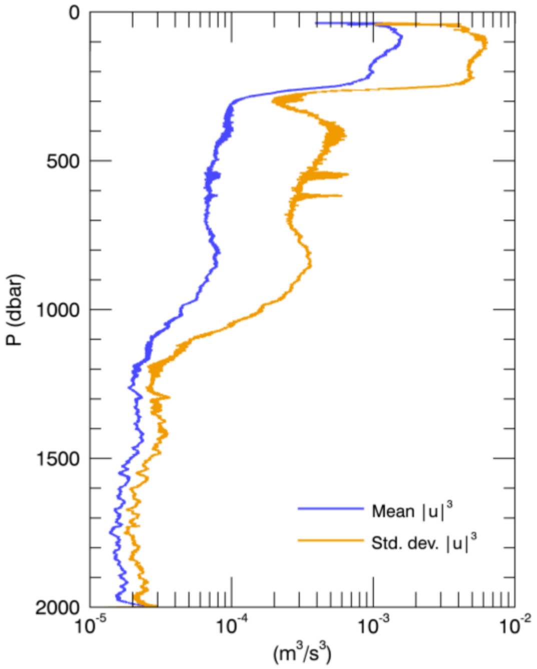

Figure: The mean (blue) and standard deviation (orange) of the current speed cubed, $|u|^3$, as a function of depth measured by MMP at BGEP mooring D between 2005 and 2017. (Note the log-scale on the x-axis).

2) the argument in favor of an increase in eddy activity, as outlined in the abstract, should be made clearer. The authors state (line 36-40) that

"The dramatic loss of sea ice and acceleration of surface currents after 2007 led to a net annual wind energy input to the BG, while the potential energy stored in the BG halocline decreased. Meanwhile, FWC remained stable despite net Ekman downwelling. To balance this, energy dissipation and FWC equilibration by oceanic eddies must also have increased relative to the ice-ocean governor mechanism after 2007."

In the geostrophic, baroclinically adjusted limit used by the authors surface currents are a diagnostic variable of potential energy so that if one increases the other one has to increase too (and viceversa). Within the assumption of constant density difference used by the authors, the same should hold for FWC too. This can be deduced by equation #1 and #3 of the paper itself: on time scales longer than a year or so, both currents and FWC are a function of the isopycnal depth anomaly. Given that the decrease in potential energy is used as the main proxy for an increase in eddy activity, what is increasing and what is decreasing should be made clearer. Incidentally, the geostrophic constrain is also the reason why kinetic energy is always two order of magnitudes smaller than potential energy, as stated in the section starting on line 390 (see also line 117-118).

The available potential energy (APE) and magnitude of surface currents are indeed diagnostic quantities but are not equivalent, i.e., they do not have to be strictly linearly related because the APE depends on the spatial pattern of the halocline depth (or alternatively on the pattern of the surface current). APE is a quadratic function of halocline depth anomaly from the state of rest, whereas the geostrophic currents are proportional to gradients of halocline depth. Thus, APE is by

our definition a ‘global’ quantity that depends on the spatial distribution of the halocline in the entire domain, whereas the geostrophic velocity is a local quantity that it depends on the local gradient of halocline depth. These distinct formulations can result in non-trivial relation between the two quantities. In classical GFD textbooks, e.g. Gill (1982), isopycnals are presented as straight lines and this fixed linear shape makes APE and geostrophic currents to be exactly linearly correlated; however, if the halocline profile deviates from the linear one and also changes its shape in time, then APE and geostrophic currents can behave differently from just being linearly correlated. This is indeed what we find in the BG evolution after ~2009: APE is decreasing while the magnitude of currents are not. We have clarified this in the revised manuscript.

3) Some additional clarification about how the energy budget is closed would be very useful. I have not tried to close it myself, but a freshwater budget should give comparable results, especially in light of the discussion of the proportionality between Ekman pumping and power input above. In addition to the freshwater comment in the abstract, the author states that (line 63-65, see also line 86-90)

"However, the time-mean area-averaged Ekman pumping across the BG is persistently negative, implying a tendency for halocline deepening and freshwater accumulation that cannot continue in perpetuity and must ultimately be balanced by other processes."

According to my own estimates (see http://mgl.mit.edu/pdf/meneghello2018exploring.pdf, currently under review), focusing on the same period studied by the authors, the mean 2003-2014 Ekman downwelling can be accounted as follows:

- 12.2 m/y of ice and wind driven downwelling (neglecting the ice-ocean feedback)
- 9.8 m/y of ice-ocean feedback driven "upwelling"

The residual 2.4m/y downwelling is taken into account by

- 1.8 m/y of eddy driven "upwelling"
- 0.6 m/y downwelling accumulated in the 7m isopycnal depth increase over 12 years.

Model- and observation-based estimates by Zhong et al. (2018) reports similar values for the residual Ekman pumping. In either case, the freshwater budget is closed as all freshwater accumulated by Ekman convergence is accounted for in the isopycnal depth increase.

This does not mean that eddy activity has not increased: as pointed out above, even the simplest eddy diffusivity closure suggests an increase in eddy flux of ~50% as a consequence of the 7m, or ~50%, increase in isopycnal depth anomaly, but this is not the argument used by the authors. Their conclusions seems to be based on the numbers presented in the paragraph starting on line 158: for 2008-2014 "there was net wind energy input of ~ 2.1 PJ/year, an APE decline of 1.7 PJ/year and a dissipation by eddies of 4.3 PJ/year". Provided the wind energy input is a good estimate (see discussion about the second assumption above), this leaves 0.5 PJ/year of energy input unaccounted for. Large scale kinetic energy is not a candidate because it is negligible. From eq. 4 I suppose the missing term is Ekman boundary flux, but then the sign is wrong. The cumulative energy associated to ekman boundary flux seems to decrease in Figure 2a, implying an energy output, not an input. I tried to ball park the numbers from Figure 2a and 2c, between 2009 and 2014 we have

- 40 PJ added by atmospheric ocean stress work
- 30 PJ removed by ice-ocean stress work
- ~1 PJ removed by ekman boundary flux

- ~2 PJ of potential energy loss

leaving ~7 PJ to be dissipated by eddies to close the balance. These numbers are consistent with the 2003-2014 Ekman pumping balance discussed before: in both the Ekman pumping and the energy budget about 75-80% of the input is taken into account by the ice, and the 14-17% by the eddies. That would correspond to an eddy dissipation of 1 PJ/y rather than the 4.3 PJ/year suggested by the authors. Mine is clearly not a rigorous analysis, but I hope it clarifies where I am confused.

As discussed above (re: 12-month smoothing) we have moved away from reporting linear trends and now discuss multi-annual changes - it seems that part of your confusion comes from our use of linear trends. From the time series in Figure 2 the system was roughly in balance before 2007. After the system was 'knocked out of balance', between 2009 and 2014, we have the following total contributions

- Wind energy input of ~9.5 PJ
- Ekman boundary flux added ~2.8 PJ
- APE decreased by ~3.2 PJ
- dissipation by eddies was ~15.7 PJ.

This balances within our uncertainties. Another way to look at this is if you sum the wind work (gray), boundary flux (pink) and dissipation (purple) time series' in Figure 2a & c, you will get the APE (orange) time series.

4) Another claim made by the authors is that eddies are required to transfer energy from the southern region, when it enters the gyre, to the northern part, where it is dissipated. A much simpler mechanism doing that is the geostrophic current itself: energy will indeed enter in the ice-free south and be dissipated in the ice-covered north, but it would be transported by the pressure field driving the current.

Good point, we have added this to the manuscript.

5) in the paragraph starting on line 243, the authors write:

"Dissipation of energy underneath sea ice will also increase as currents speed up, however, as sea ice continues to decline for longer periods of the year, its cumulative capacity to dissipate upper open energy will diminish".

Energy dissipation varies linearly with the ice cover, but with the cube of the current speed, as pointed by the author themselves on one 177. This conclusions is not as obvious as it seems.

We have clarified this passage of text in the revised manuscript.

In conclusion, while I do agree with the idea that i) eddies are critical processes fo the accurate representation of the BG systems in models, ii) that eddy activity has probably increased after 2007, and iii) that closing the energy budget has indeed the potential to show it, I suggest a more detailed is necessary to show that eddy activity "must have increased".

We hope that our replies to your comments have alleviated some of your concerns about our approach and conclusions. Again, we feel that providing an observational estimate, at the gyre-scale, the increasing role of mesoscale eddies since 2009 in balancing the energy budget of the Beaufort Gyre is a novel and important result. We appreciate the reviewer flagging a number of areas where there is the potential for misinterpretation of these results, but we feel that we have addressed all of these issues

candidly in the revised text. The manuscript has been strengthened substantially by this review process and we hope the reviewer finds this study suitable for publication.

Other minor comments below:

line 114: no model or layers have been mentioned before this point

Clarified to just say we estimate “the density difference across the halocline”.

line 151-152: The author states that "However the eddy response may lag the forcing by a few years [Manucharyan et al., 2017].". But Manucharyan et al. 2017 studies the response of the entire Beaufort Gyre, not the response of the eddy themselves. The eddies respond to a change of the isopycnal slope on the time scale of baroclinic instability, which should be a couple of weeks.

You might be referring to Manucharyan et al. (2016), where there is indeed no discussion of the lag. However, here we are referring to Manucharyan et al. (2017) that solely addresses the issue of the lag between eddy kinetic energy (and the eddy streamfunction) and isopycnal slope in the context of the BG. The lag could be up to a few years in the idealized simulations of the BG that include continental slopes. The crucial role of continental slopes is that they are regions of reduced eddy diffusivity and have a Eulerian mean overturning that is dramatically different from the Ekman overturning (see Manucharyan and Isachsen (2019)). The lagged eddy response originates mainly near continental slopes then propagates into the interior and affects the evolution of the overall freshwater content. This is a relatively minor discussion point of our manuscript and our data does not allow us to diagnose if this lag exists, yet we think that it might be important for interpreting the time evolution of the eddy energetics and magnitude of BG currents when future studies would address interannual variability of eddy kinetic energy and eddy diffusivity.

line 236-242: the ice-ocean governor is not needed to balance the Ekman pumping if the Ekman pumping itself is upwelling instead of downwelling. The same is true for the eddies.

We have removed discussion of the governor/eddies in this passage as it was not really relevant for our point, which is that 1) we expect a switch to cyclonic circulation would lead to freshwater release and spin-down of the BG, and 2) that the BG will become more susceptible to atmospheric forcing as more sea ice is lost.

Figures: many panels show work with units of Watts. Power is measured in Watts, work in Joules.

We have updated the figures and captions where appropriate.

Equation 6: Ai is not defined

Corrected.

line 333: the values of Cdao and Cdio used should be made explicit

Corrected in the revision.

line 500: this citation is repeated (and some dois seems wrong in many other citations)

We have checked through the citations in the revision.

======================

Bibliography

E. W. Doddridge, G. Meneghello, J. Marshall, J. Scott, C. Lique (2019) A Three-way Balance in The Beaufort Gyre: The Ice-Ocean Governor, Wind Stress, and Eddy Diffusivity. J. Geophys. Res. C: Oceans, doi:10.1029/2018JC014897

G. Meneghello, J. Marshall, J.-M. Campin, E. W. Doddridge, M.-L. Timmermans (2018a) The Ice-Ocean governor: ice-ocean stress feedback limits Beaufort Gyre spin up. Geophys. Res. Lett., 45. doi:10.1029/2018GL080171

Roquet, Wunsch and Madec (2011) On the Patterns of Wind-Power Input to the Ocean Circulation JPO https://doi.org/10.1175/JPO-D-11-024.1

Zhao, M., M.-L. Timmermans, S. Cole, R. Krishfield, and J. Toole (2016), Evolution of the eddy field in the Arctic Ocean's Canada Basin, 2005–2015, Geophys. Res. Lett., 43, 8106–8114, doi 10.1002/2016GL069671

Zhong, W., Steele, M., Zhang, J., & Zhao, J. (2018). Greater role of geostrophic currents in Ekman dynamics in the western Arctic Ocean as a mechanism for Beaufort Gyre Stabilization. Journal of Geophysical Research: Oceans, 123, 149–165.https://doi.org/10.1002/2017JC013282

Additional comments on "Enhanced eddy activity in the Beaufort Gyre in response to sea ice loss" by T. Armitage et al.

In addition to my already submitted review, I was asked by the editor to comment on the concerns of reviewer 1.

While I agree with reviewer 1 that large part of the methods used have been previously discussed, and that similar conclusions has been published in recent work, large-scale observational evidence of the evolution of the eddy field in the Arctic is indeed missing and will be very welcome by me and, I think, by the Arctic Oceanography community in general. I am convinced that the topic addressed is very current, and that a paper covering it should be considered for publication.

At the same time, I partly agree with reviewer 1 when he states that "I found only speculation that reduction of APE is mainly associated with generation of eddies". This might be too strong a statement: closing the energy budget is a very welcome attempt to ground such speculation in observations. What remains speculative is the idea that the terms considered in the energy budget cover everything: other components of the energy budget are quickly dismissed by the authors with very little discussion.

> We have attempted to constrain all parts of the energy budget for which we have appropriate observations, and of course we can only estimate the relative importance of other terms in the energy budget that we cannot observe. While we acknowledge that uncertainties exist in all the observable variables, our energy budget approach does provide valuable insights into the BG dynamics that are subjected to further validation. Specifically, our results suggest that a specific change in eddy activity might have occurred and this result could be further tested with different observations and realistic models. We have added the statement to the revised manuscript that our results present a hypothesis since they are based on an estimate of the energy budget.

An example is energy dissipation by bottom flow, see discussion in my last review. These might be indeed negligible on long time scales --- corresponding to an equilibrated gyre --- but they are most probably not at the short time scales discussed here. More importantly there is a mismatch between the daily time scale at which the energy input is computed, and the long time scale at which the energy budget in its current form can be considered closed.

> See the Figure above and our reply about energy dissipation by bottom flow. We have estimated the (relative) dissipation as a function of depth using mooring velocity data and confirmed that the bottom dissipation (even at daily time-scales) is significantly smaller than the dissipation under ice. To confirm the energy calculations are reasonable, we consider the rapid APE changes that followed the strong energy input in 2007-08: from our calculations the magnitude of these major terms in the energy budget *d(APE)/dt* and $\tau_o \cdot u_g$ for this specific year are consistent. The APE calculations use independent data from $\tau_o \cdot u_g$ and hence the achieved consistency provides further support that calculating domain-averaged $\tau_o \cdot u_g$ is indeed a useful predictor of the energy changes in the BG.

Concerning the estimation of APE, I agree with reviewer 1 that it is not clear how halocline depth and FWC are stable or increasing after 2007, but APE decreases. In the framework used by the authors, I would have expected the three to be closely related, and the text is not clear on why this is not the case (see discussion in my last review).

> We have addressed the point that APE and FWC are not equivalent quantities and there is no

formal mathematical requirement for these two quantities to be linearly correlated. Please see discussion of this point in the reply to your comment above.

Summarizing, while I do think that the topic addressed by this study is of interest and deserves publication, and that closing an energy budget is an interesting approach to the problem, I also think a better justification and explanation of the hypothesis underlying the energy budget, or a modification of the budget itself, would be needed to address the concerns of reviewer 1 (and which in large part I share).

We hope that we have clearly addressed all your comments and have included appropriate discussion of the limitations in the paper. If you have any specific comments regarding the assessment of reviewer #1 we would be happy to address them.

I hope my comments are helpful.

Thank you for your detailed and helpful review, which has led to more accurate discussions in the paper that better highlights the limits of the paper.
